# Development of a 3D Coupled Physical-Biogeochemical Model for the Marseille Coastal Area (NW Mediterranean Sea): What Complexity Is Required in the Coastal Zone?

**DOI:** 10.1371/journal.pone.0080012

**Published:** 2013-12-04

**Authors:** Marion Fraysse, Christel Pinazo, Vincent Martin Faure, Rosalie Fuchs, Paolo Lazzari, Patrick Raimbault, Ivane Pairaud

**Affiliations:** 1 IFREMER, Laboratoire Environnement Ressources Provence Azur Corse, La Seyne sur Mer, France; 2 Aix Marseille Université, CNRS/INSU, IRD, Mediterranean Institute of Oceanography (MIO), UM 110, Marseille, France; 3 Université de Toulon, CNRS/INSU, IRD, Mediterranean Institute of Oceanography (MIO), UM 110, La Garde, France; 4 Dept. of Oceanography, Istituto Nazionale di Oceanografia e di Geofisica Sperimentale (OGS), Trieste, Italy; University of Vigo, Spain

## Abstract

Terrestrial inputs (natural and anthropogenic) from rivers, the atmosphere and physical processes strongly impact the functioning of coastal pelagic ecosystems. The objective of this study was to develop a tool for the examination of these impacts on the Marseille coastal area, which experiences inputs from the Rhone River and high rates of atmospheric deposition. Therefore, a new 3D coupled physical/biogeochemical model was developed. Two versions of the biogeochemical model were tested, one model considering only the carbon (C) and nitrogen (N) cycles and a second model that also considers the phosphorus (P) cycle. Realistic simulations were performed for a period of 5 years (2007–2011). The model accuracy assessment showed that both versions of the model were able of capturing the seasonal changes and spatial characteristics of the ecosystem. The model also reproduced upwelling events and the intrusion of Rhone River water into the Bay of Marseille well. Those processes appeared to greatly impact this coastal oligotrophic area because they induced strong increases in chlorophyll-a concentrations in the surface layer. The model with the C, N and P cycles better reproduced the chlorophyll-a concentrations at the surface than did the model without the P cycle, especially for the Rhone River water. Nevertheless, the chlorophyll-a concentrations at depth were better represented by the model without the P cycle. Therefore, the complexity of the biogeochemical model introduced errors into the model results, but it also improved model results during specific events. Finally, this study suggested that in coastal oligotrophic areas, improvements in the description and quantification of the hydrodynamics and the terrestrial inputs should be preferred over increasing the complexity of the biogeochemical model.

## Introduction

Coastal regions, located at the interface between oceanic and terrestrial systems, play a crucial role in earth system functioning [1]. Greater than 60% of the world's population lives less than 60 km from the sea, increasing the human pressure on these systems [2]. Understanding the fate of anthropogenic inputs from major cities and their impacts on the adjacent marine ecosystems is essential for the protection and management of coastal waters. Although it is clear that coastal systems are locally strongly impacted by human activities, it remains difficult to distinguish between climatic and anthropogenic forcing [1]–[3]. Modeling approaches can assist with this difficulty and are useful tools for studying such a complex coastal environment.

Marseille is the second largest city in France, and the metropolitan area of Marseille extends beyond the city limits with a population of 1 038 940 and a density 1 718 people per km^2^ [4]. The density of contaminant-generating industries in the city of Marseille and the quantity of sewage are highly representative of large, modern Mediterranean cities. Hydrodynamic and biogeochemical processes affect the transport and form of chemical compounds; for example, contaminant speciation often depends on suspended matter and on organic compounds (Particulate Organic Carbon (POC) and Dissolved Organic Carbon (DOC)) [5]. Marseille was thus chosen for the development of a numerical tool (a chain of models) to assess the raw inputs (from city to sea) and exports (from mid-sea to open sea) of chemical contaminants. This tool was developed based on the coupling of a hydrodynamic model [6], a sedimentary model, a biogeochemical model and a model of chemical contamination. This paper presents the coupled hydrodynamic-biogeochemical compartment.

**Figure 1:**
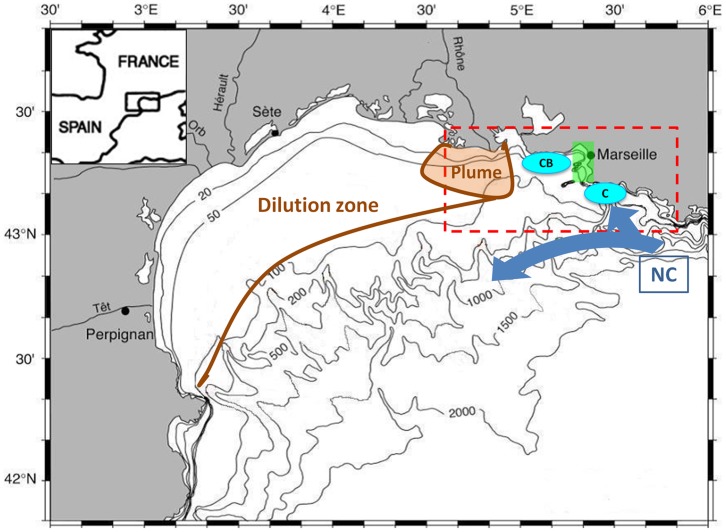
Map of the Gulf of Lion and the model domain. The RHOMA model domain (red dotted lines), the Bay of Marseille (green rectangle), the “Cote Bleue” and “Calanques” upwelling spots (blue circles), the Rhone River plume and intrusion zone (in brown) and the Northern current (NC) are represented.

The Marseille coastal area is located in the eastern part of the Gulf of Lions (GoL) in the western Mediterranean Sea (Figure 1). The GoL is one of the most productive areas of the Mediterranean Sea [5], even if the Mediterranean Sea remains oligotrophic. The biogeochemical functioning of the GoL is strongly impacted by inputs from the Rhone River as it is the most significant source of freshwater and nutrients in the Mediterranean Sea; these inputs have a direct influence on the primary production. On an annual basis, approximately 50% of the primary production in the GoL can be attributed to terrigenous inputs [7], [8]. The hydrodynamics of the GoL are complex and highly variable [9] because there is strong temporal and spatial variability in the forcing occurring at the eastern part of the GoL [10].

The biogeochemical functioning of the Bay of Marseille (BoM) is complex and highly driven by hydrodynamics. The hydrodynamics of the Marseille coastal area were studied in details using a modeling approach [6]. The primary forcing components are the two dominant winds (north-northwesterly winds, which favor upwelling, and southeasterly winds, which favor downwelling) and the oligotrophic Northern Current (NC), which flows along the continental slope toward the west [11] and occasionally intrudes on the shelf [6], [12]–[14]. North-northwesterly wind gusts induce upwelling zones off the “Cote Bleue” and off “Calanques” [6], [11]. During an upwelling event, cold, rich waters are advected upwards [15], which can induce (in favorable cases) an increase in primary production. A case study undertaken in the BoM [16] showed that primary production tripled at a coastal station influenced by upwelling compared with a reference offshore station. Observation [17] and modeling studies of the Region Of Freshwater Influence (ROFI) [18]–[20] show the predominant westwards direction of the Rhone River plume. A less common orientation of the Rhone River plume, toward the East up to 40 km from the Rhone River mouth and offshore in the BoM, was recently observed [21]. The presence of water from the Rhone River in the BoM was established in a modeling study by Pairaud et al. [6]. The effects of the eastward intrusion events of the Rhone River plume were observed on biological production, local phytoplankton blooming and chromophoric dissolved organic matter (CDOM) production was measured [22]. The BoM is also impacted by urban rivers, a Wastewater Treatment Plant (WWTP) and atmospheric deposition, but their impacts on the biogeochemical functioning of the Bay and nutrient limitation remain to be studied.

The knowledge available on nutrient limitation is at the scale of the Mediterranean Sea and its Western Basin. Thus, it is essential to understand how photosynthetic production is or is not limited by nitrogen (N) and phosphorus (P) because the cycles of key nutrient elements, such as N and P, have been massively altered by anthropogenic activities [23]. In the Mediterranean Sea, N or P concentrations are generally considered to be limiting factors for algal production [24]. Van Wambeke et al. [25] confirm that P limitation of bacterioplankton is a generalized phenomenon in the Mediterranean Sea. Indeed, with a NO_3_∶PO_4_ ratio of 65–80 [26], [27], the Rhone River contributes to the relative P deficit of the Mediterranean Sea [5]. Atmospheric inputs also provide N in excess relative to P (mean DIN/DIP ratio of 60 in the Western Mediterranean Basin) and contribute to the P limitation [28]. In addition, in the northwestern Mediterranean Sea, P limitation for both phytoplankton and bacteria was suggested by Thingstad et al. [29]. Therefore, a modeling approach could aid in the understanding of the limitation functioning of the Marseille coastal area.

3D physical and biogeochemical modeling approaches were used in the GoL to study upwelling [30], nitrate fluxes between the margin and the open sea [31], the functioning of the planktonic ecosystem during spring and its impact on particulate organic carbon deposition [32] and the biogeochemical functioning of eddies [33]. Recently, Fontana et al. [34] performed assimilation of chlorophyll-a remote sensing data in a 3D coupled physical-biogeochemical model for the area near the mouth of the Rhone River. This study represents the first 3D, high-resolution coupled physical-biogeochemical modeling study of the BoM. Coastal zones are highly variable both hydrodynamically and biogeochemically; therefore, the modeling approach cannot be the same as used in the open sea. Franks [35] strongly suggested that the model should be designed around the scientific question being addressed. Indeed, the choice of the complexity of the biogeochemical model usually depends on the study to be conducted [36]. In our case, a model light in computational cost has many advantages for operational applications and for coupling it with other models (sediment and contamination models). Moreover, the complexity of 3D coupled models can make them particularly difficult to evaluate. Arhonditsis and Brett [37] performed a systematic analysis of 153 biological models that incorporated plankton. Only 47% of the models assessed had any performance-related accuracy assessment, and only 30% determined some measure of goodness of fit with respect to observed values. In this paper, we attempted to develop the simplest model to address our questions and then to assess its ability to reproduce both temporal and spatial variability and the primary processes of this coastal ecosystem.

The objective of this study was to develop and to assess a physical-biogeochemical coupled model to characterize the biogeochemical functioning of the Marseille coastal zone. An initial version of the coupled model was developed, and then the biogeochemical model was improved by adding the P cycle. Then, we assessed both versions of the model and whether the addition of the P cycle yields an improvement in the model simulation. In the final section, we discuss the complexity required in coastal ecosystem modeling and the contribution of model results to the understanding of the Marseille coastal area.

## Materials and Methods

### 1. Models

#### 1.1. The hydrodynamic model: MARS3D RHOMA

The hydrodynamic model used for this study was the free surface, three-dimensional MARS3D model (3D hydrodynamic Model for Applications at regional Scale, IFREMER). The high resolution MARS3D-RHOMA configuration was applied and validated to the forecast of the oceanic circulation off Marseille [6], with a horizontal resolution of 200 m and 30 sigma vertical levels. The time step was fixed at 30 s. Atmospheric forcing, hydrodynamic open boundary conditions and the numerical schemes of the model were described by Pairaud et al. [6]. The model grid resolution used in this work is a downgrade of the version described in Pairaud et al. [6], with a 400-m horizontal resolution. Similar validation was performed on the 400-m version, and the processes were well reproduced.

#### 1.2. Coupled model

The coupled model domain covered an area of 100 km×48 km between the mouth of the Rhone River and Cape Sicié. The study area was discretized horizontally using a uniform mesh of 252×120 cells at 400-m resolution. The vertical direction was divided into 30 sigma levels (refining the resolution close to the surface and bottom).

The hydrodynamic model and the biogeochemical model were coupled online (Equation 1). The biogeochemical model calculated all variables tendency (Source-Minus-Sinks, SMS) for each grid point every 20 minutes, whereas the physical model performed the advection-diffusion of the biogeochemical concentrations (C) with a 30-second time-step. The analytical formulations of the advection and diffusion terms are the same as those for the temperature and salinity in the physical model.
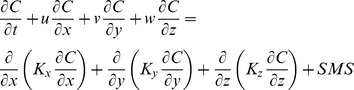
(1)


The coupling was introduced as the extinction light coefficient calculated by the biogeochemical model used in the function of the chlorophyll-a concentration, which induced a retro-action on the temperature calculation of the physical extinction light coefficient; thus, it was truly a coupled online model. Total radiance was read from the meteorological file (MM5) by the hydrodynamic model.

#### 1.3. The biogeochemical model: ECO3M MASSILIA

The biogeochemical model was implemented using the Eco3M (Ecological Mechanistic and Modular Modeling) modeling platform [38], [39]. A new biogeochemical model (ECO3M-MASSILIA) was developed for this study. The model structure used is primarily based on the pelagic plankton ecosystem model published by Faure et al. [40], [41] but without the P cycle.

The biogeochemical model was split into 5 compartments (phytoplankton, heterotrophic bacteria, dissolved and particulate organic matter and dissolved inorganic matter). This model allowed for a variable intracellular content of phytoplankton and bacteria. Another particularity was that the zooplankton were not represented as a state variable of the model; rather, their physiological fluxes (e.g., grazing or excretion) were considered as explicit functions. The closure formulation of the model was based on the assumption that all of the matter grazed by the zooplankton and the higher trophic levels was returned to one of the pools of organic or inorganic matter (dissolved and particulate organic matter and dissolved inorganic matter).

**Figure 2:**
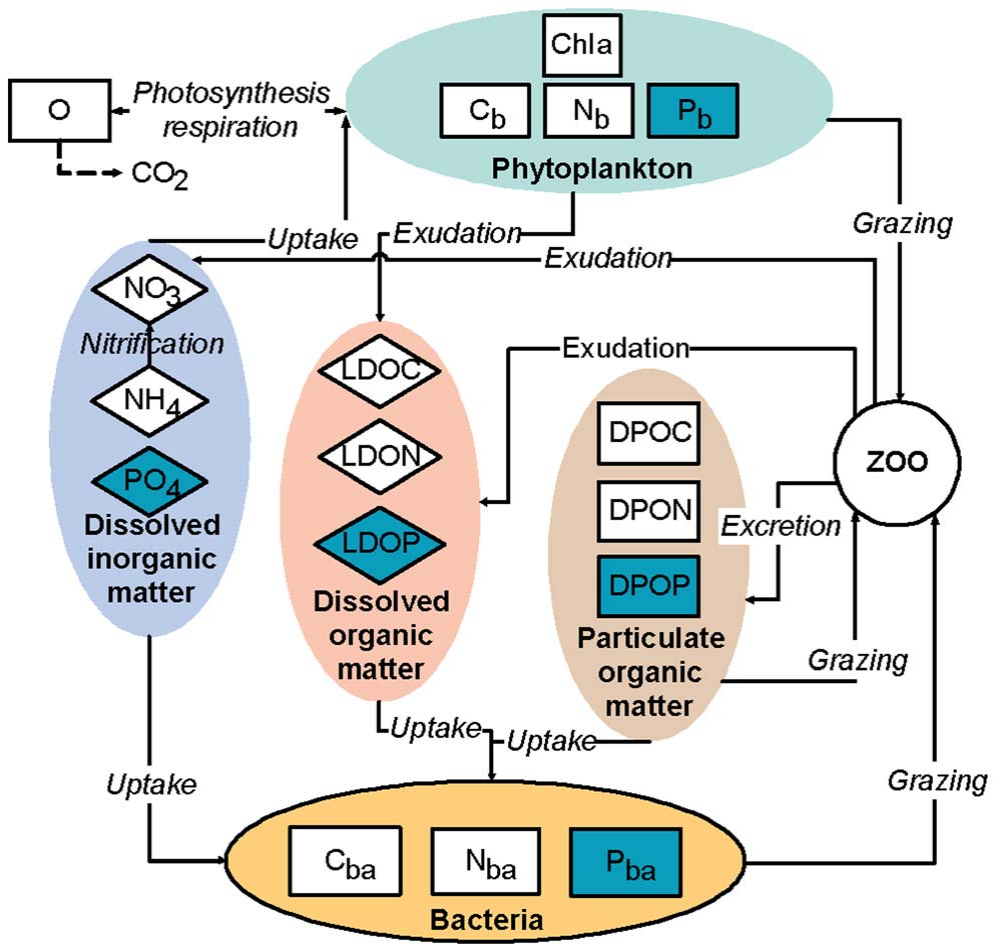
The pelagic biogeochemical model. The ECO3M-MASSILIA-noP (only white-colored variables) and ECO3M-MASSILIA-P (white- and purple-colored variables) versions of the biogeochemical model are represented.

In this paper, we present and discuss only the modifications applied to the initial version of the model. First, the ECO3M-MASILLIA-noP was developed with new parameterization adapted for the Mediterranean Sea and the addition of grazing limitation by temperature. Then, the ECO3M-MASILLIA-noP was modified with the addition of the P cycle (ECO3M-MASILLIA-P). Thus, the pelagic ecosystem was summarized in 12 or 17 state variables (Table 1), and the cycles of C and N were described by the ECO3M-MASSILIA-noP, with the addition of P for the ECO3M-MASSILIA-P (Figure 2). We refer the reader to Annex S1 for the detailed equations of the biogeochemical model.

**Table 1:** Biogeochemical model variables (ECO3M-MASILLIA-P version).

	Variable	Definition
1	C_B_	Phytoplankton Carbon
2	N_B_	Phytoplankton Nitrogen
3	Chl.a	Phytoplankton Chlorophyll-a
4	CBA	Bacterial Carbon
5	NBA	Bacterial Nitrogen
6	DPOC	Detrital Particulate Organic Carbon
7	DPON	Detrital Particulate Organic Nitrogen
8	LDOC	Labile Dissolved Organic Carbon
9	LDON	Labile Dissolved Organic Nitrogen
10	NO3	Nitrates
11	NH4	Ammonium
12	O	Oxygen
13	PB	Phytoplankton Phosphorus
14	PBA	Bacterial Phosphorus
15	DPOP	Detrital Particulate Organic Phosphorus
16	LDOP	Labile Dissolved Organic Phosphorus
17	PO4	Phosphorus

Only variables from 1 to 12 for ECO3M-MASILLIA-noP version. Units in µmol.L^−1^ except Chlotophyll-a which is in µg.L^−1^.

The parameterization of the biogeochemical model was adapted for the Marseille coastal area (Annex S2). Previous studies of the phytoplankton community in the Marseille – Rhone area concluded that diatoms dominated the phytoplankton community [42], [43]. When possible, phytoplankton parameters were chosen to represent diatoms. For the purpose of parameter refinement, we implemented zero-dimensional models, which allowed us to perform many simulations with different parameters sets.

A coastal study in the northwestern Mediterranean Sea demonstrated that the grazing rate had strong seasonality: the grazing is lower in winter and higher in summer [44]. Another study reported that the development of the zooplankton was impacted by the water temperature [45]. This seasonality was introduced into the model as a function linking the grazing rate to the temperature (T) (Equation 2). The limitation function f(T) [46] was applied to the grazing rate.
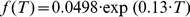
(2)


The literature on the limitation by P [25], [29] and the potential deficiency in P of diverse inputs into the study area [26]–[28] highlight the importance of considering this element. For the addition of the P cycle in ECO3M-MASSILIA-P, five supplementary states variables were added (Figure 2) : dissolved inorganic phosphorus (PO_4_), labile dissolved organic phosphorus (LDOP), detrital particulate organic phosphorus (DPOP), phosphorus phytoplankton biomass (Pb) and phosphorus bacterial biomass (Pba). For coherence, the representation of the P cycle mimicked that of the N cycle. The structural modifications are described hereafter for the P equations that differed from those of the N. The full model equations are detailed in Annex S1.

Concerning the phytoplankton compartment, the phytoplankton biomass in phosphorus (Pb) was introduced as a state variable. Pb depends on the PO_4_ uptake and zooplankton grazing (Equation 3).
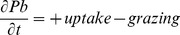
(3)


The equations of the phytoplankton biomass in carbon and chlorophyll were modified. The carbon biomass equation was modified through the term for the limitation of the phytoplankton maximal growth rate (P^C^_m_) by nutrient (Q*) (Equation 4). The co-limitation of phytoplankton by nutrients was introduced into the equation for phytoplankton carbon biomass as an independent nutrient co-limitation of type I [47]. The co-limitation between N and P was calculated using the Liebig's Law of the minimum [48], [49]. The phytoplankton limitation by P was calculated in the same manner as by N: with the intracellular quota (Q) and the Geider formulation [50]. Consequently, phytoplankton production is controlled by the most limiting internal ratio (Q*).
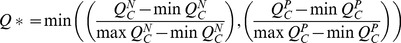
(4)


The phytoplankton chlorophyll-a concentration was a diagnostic variable related to the phytoplankton biomass by carbon (C_B_) and the variable ratio Chla∶C (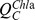) (Equation 5). Equation 6 describes the 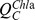 of phytoplankton depending on the phytoplankton N-to-C ratio and the limitation by the most limiting internal ratio (Q*). As Q* decreases toward zero, 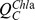 also decreases, as does the limitation on Chla. In Smith and Tett [51] and Faure et al. [52], the variable Q* only represented the internal N∶C ratio. To also consider the limitation of chlorophyll-a production when phytoplankton was limited by P, we modified the equation for Q* (Equation 4).
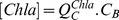
(5)

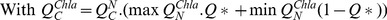
(6)


As for phytoplankton, heterotrophic bacteria were described in terms of carbon, nitrogen and phosphorus. To include the P cycle in the microbial loop, the bacterial biomass in phosphorus (Pba) was added as a new state variable. Pba varied with the uptake and grazing of organic matter (DPOP and LDOP) and inorganic matter (PO_4_) (Equation 7).
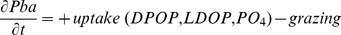
(7)


We simulated the temporal evolution of heterotrophic bacteria according to the cell quota theory. This concept [53] considers that growth rate (μ^BA^_max_) is a function of limiting nutrients within the cell. A new system of co-limitation by C, N and P was needed to control the bacterial production (BP) (Equation 8). We choose to represent the co-limitation (f) of the bacterial production by the Liebig's law (Equation 9), similarly to the representation of phytoplankton production. However, even if Liebig's law was used for the co-limitation of the phytoplankton, the limitation of the maximum growth rate (μ^BA^_max_) of bacteria was calculated differently. 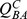, 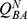 and 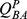 represents the carbon, nitrogen and phosphorus cell quota of bacteria (see Annex S1 for more details). As the ratio (min 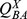)∶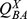 increased, the limitation of the bacteria by the element X also increased. The most restrictive element corresponds to the higher ratio (min 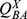)∶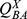.
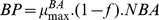
(8)

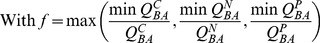
(9)


#### 1.4. Modeling strategy

To determine the spin-up period of the model, tests were conducted for the year 2007 by replacing winter initial conditions with summer initial conditions. We considered the maximum spin-up period to be completed when all state variables were equal between the two runs. The period lasted 90 days; therefore, the model results can be studied from April 2007 onwards (Annex S3).

The hydrodynamic open boundary conditions and initial conditions of the MARS3D-RHOMA configuration are described in Pairaud et al. [6]. The biogeochemical open boundary conditions and initial conditions were built from a coupled model applied to a larger area (GoL) at a horizontal resolution of 1.2 km and 30 vertical sigma levels. The hydrodynamic model (MARS 3D-GOL configuration) was based on the MARS3D-MENOR configuration validated by Nicolle et al. [54]. The MARS 3D-GOL configuration was coupled online with the biogeochemical ECO3M-MASSILIA-P [55]. The biogeochemical open boundary conditions of this larger model were provided by the OPATM-BFM pre- and operational runs 2007–2011 performed in the framework of the Mersea and MyOcean projects [56].

Atmospheric dry deposition (dry and wet deposits) was sampled weekly by the national MOOSE program (Mediterranean Oceanic Observing System on Environment) and the “Service d'Observation” of the Mediterranean Institute of Oceanography (S.O. MIO). Inputs from rain (wet deposition) were measured for each rain event. The device (collecteur MTX-Italia) was installed on an island located in the bay off Marseille. Atmospheric samples were analyzed for soluble components (nitrate (NO_3_), ammonium (NH_4_), phosphate (PO_4_), dissolved organic carbon (DOC), dissolved organic nitrogen (DON) and dissolved organic phosphorus (DOP)) and insoluble particulate matter (particulate organic carbon (POC), particulate organic nitrogen (PON) and particulate phosphorus (POP)). Wet atmospheric concentrations were applied to the rainfall represented by the atmospheric model MM5. Dry deposition was applied as a mean flux between each pair of sample dates.

Between 2007 and 2011, daily averaged Rhone River discharges at the Beaucaire station were available and provided by the “Compagnie Nationale du Rhone”. The “Grand Rhone” located in the modeling domain represents only 90% of total Rhone River discharge [57], [58]. Marseille has 4 main Urban Rivers (Aygalade, Belvedère-Figuière, Huveaune-Jarret and Bonneveine) and a Wastewater Treatment Plant (WWTP), which flows in at Cortiou (Annex S4). The Marseille Urban Rivers discharges were available with a time step of 6 minutes, and the daily discharge from the Marseille WWTP was available (Data provided by the DEA-MPM (Direction de l'Eau et de l'Assainissement-Marseille Provence Métropole)). The Berre Lagoon, which is a shallow semi-confined ecosystem, is connected to the Mediterranean Sea via the Caronte channel. The latter was represented in the coupled model as a river with a constant discharge of 20 m^3^.s^−1^. River and WWTP concentrations.

Biogeochemical concentrations in the Rhone River (NO_3_, NH_4_, PO_4_, POC, PON, POP, DOC, DON and DOP) were measured daily and provided by the national MOOSE program and the SO MIO. As our model considered only the labile fraction of organic matter, a percentage representing the labile fraction of organic matter was applied to the daily Rhone River concentrations. However, studies of the lability of Rhone River organic matter were rare. The labile fraction of particulate organic carbon (DPOC) was considered to be 18% of POC [59]. Déliat [60] estimated that approximately 20% of the Rhone River DOC was biodegradable; thus, we computed LDOC as equal to 20% of DOC. The labile fraction of DON (LDON) was also estimated to be 20% of DON [60]. Experiments in Loch Creran (Scotland) showed that bioavailable DOP (BDOP) accounted for 88±8% of DOP (average±SD) [61]. The same percentages were applied to the particulate organic matter concentrations to obtain DPON and DPOP.

The concentrations in the urban rivers were not always available from in-situ data for all of the variables and hence were derived from empirical relationships (Table 2). The Rhone River concentrations (described above) were applied to the concentrations of nutrients in other Marseille Urban Rivers. Organic matter concentrations in the Marseille Urban Rivers and from the WWTP were deduced from suspended particulate matter (SPM) concentrations [62] with constant ratios. The same hypotheses on the lability of the Rhone River organic matter were applied to the urban river concentrations. The concentrations of NH_4_ and NO_3_ from the Marseille WWTP were available daily (Data provided by the DEA-MPM (Direction de l'Eau et de l'Assainissement-Marseille Provence Métropole)). The PO_4_ concentration was fixed to 13.4 µmol (Faure, comm. Pers.). Marine phytoplankton and bacteria species were considered to be absent from all of the rivers and WWTP inputs. The Caronte concentrations were fixed at constant values (Table 2) obtained by averaging the measured concentrations [63].

**Table 2:** Concentrations of River inputs (µmol.L^−1^).

River	NO_3_	NH_4_	PO_4_	LDOC	LDON	LDOP	LPOC	LPON	LPOP
Rhone	data	data	data	0.2*DOC	0.2*DON	0.88*DOP	0.18*POC	0.2*POC	0.88*POP
Caronte	0.75	2.72	0.4	45	3.73	0.11	1.8	1.36	0.078
WWTP	data	data	13.4	135	17	3	8403	864	372
Bonneveinee	RR	RR	RR	52.4	6.62	1.17	667	68.60	29.6
Huveaune	RR	RR	RR	38.5	4.86	0.86	869	89.4	38.57
Emiss 1	RR	RR	RR	553	69.9	12.37	2430	250	107.8
Emiss 2	RR	RR	RR	14.4	1.82	0.32	f(RD)	f(RD)	f(RD)
Aygalade	RR	RR	RR	52.4	6.62	1.17	f(RD)	f(RD)	f(RD)
Belvedere	RR	RR	RR	52.4	6.62	1.17	f(RD)	f(RD)	f(RD)

RR: Rhone River daily measured concentrations; f(RD) : concentrations linked to urban rivers discharges.

### 2. Observational data sets

#### 2.1. In-situ data

To validate the coupled model, we used a long time series of hydro-biogeochemical data collected twice monthly at the Somlit station (43°14.30′N; 5°17.30′E) located in the BoM (Annex S4). High vertical resolution profiles of temperature, salinity and oxygen were obtained between 0 and 55 m using a conductivity temperature-depth-oxygen profiler (CTDO, Seabird 19+). Water samples were collected at 3 depths with hydrological Niskin bottles for the determination of inorganic and organic nutrients concentrations. Samples for nitrate, nitrite and phosphate were collected into 60 ml polyethylene nutrients flasks and were frozen at −20°C until analysis at laboratory according to Aminot and Kerouel [64]. Samples for acid silicic were collected in 60 ml polyethylene flasks and stored at 5°C until analysis at laboratory according to [64]. Samples for ammonium determination were collected in triplicate in 60 ml polycarbonate tubes. The reagent was immediately added to the tubes and ammonium level was determined by fluorometry according to Holmes et al. [65]. Particulate organic carbon (POC) and particulate organic nitrogen (PON) in suspended matter collected on Whatman GF/F glass micro-fibre filters pre-combusted for 4 h at 450°C, were determined by using the high combustion method (1000°C) on a CN Integra mass spectrometer [66]. Chlorophyll concentrations were estimated by fluorometry [67] on suspended matter collected on Whatman GF/F filter. The data were provided by the SOMLIT network (Service d'Observation en Milieu Littoral, http://somlit.epoc.u-bordeaux1.fr).

#### 2.2. Satellite remote sensing data

We also compared the model-predicted fields of surface chlorophyll-a concentrations with the corresponding remote sensing-derived concentrations. The MODIS (Moderate Resolution Imaging Spectroradiometer) and MERIS (MEdium Resolution Imaging Spectrometer) ocean color sensors have a spatial resolution of approximately 1 km, which is more course than the spatial resolution of our model (400 m). For the comparisons, we used the remotely sensed chlorophyll-a concentration processed with the algorithm OC5 [68], [69] from the IFREMER (Institut Français de Recherche pour l'Exploitation de la Mer) database. The OC5 method [68] is empirical and derived from the OC4 algorithm of NASA (or OC3M-547 for MODIS and OC4E for MERIS). This method gives results similar to OC4 in open waters but provides more realistic values over the continental shelf [70]. The quality of the remote sensing data was evaluated in Annex S5. The remote sensing data contained observational errors, which was also the case for the in-situ data. Nevertheless, the large number of existing data, their availability throughout the year and their spatial coverage make remote sensing data essential to the consideration of spatial patterns and gradients. Thus, the comparison with the model results had to consider the observational error.

### 3. Statistical comparison with observations

To assess the accuracy of the 3D coupled online model, we used statistics highlighting correspondences between the model results (M) and in-situ and remote sensing observations (O). The study of model performance often requires multiple stages of analysis, the evaluation of the ability of the model to reproduce instantaneous station values (and trends) and the ability of the model to recreate spatial characteristics (and trends) of processes [71]. Stow et al. [72] explained that model start to have skill when the observational and predictive uncertainty halos overlap; in the ideal case, the halos overlap completely. Thus, accuracy assessment requires a set of quantitative metrics and procedures for comparing model output with observational data in an appropriate manner for the particular application. Here, we used different statistical indicators [72], [73] :

The percentage model bias (PB, model error normalized by the observations) measures whether the model underestimated or overestimated the observations [74]. The closer to zero the value is, the better the model.
The cost function (CF) gives a non-dimensional value that is indicative of the “goodness of fit” between two sets of values [73]. Radach and Moll [75] proposed an interpretation of the values of the cost functions adapted from the OSPAR Commission [76] rating. Model results are classified as very good (cost function between 0 and 1), good (1 and 2), reasonable (2 and 3) and poor (higher than 3) [77].
The correlation coefficient (R) is a measure of the strength and direction of the linear relationship between two sets of values [74].
The average absolute error (AAE) quantifies the magnitude rather than the direction of each discrepancy (Equation 10). The closer to zero the value is, the better the model.
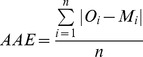
(10)
The Root Mean Square Deviation (RMSD) is also a measure of the difference between values predicted by the model and the values actually observed from the environment [74]. The RMSD has the same units as the quantity being evaluated.

To compare the variables, we used signed, normalized, unbiased RMSD (snuRMSD) to quantify the magnitude direction of each discrepancy and the normalized bias to quantify the direction of each discrepancy [78]. Those indicators are used to construct target diagrams [78].

Model developments and parameterization were performed for the years 2007 and 2008, and then the observations available for the years 2009, 2010 and 2011 were used only to assess the accuracy of both versions of the model.

## Results

We assessed the ability of the coupled model to reproduce the main characteristics of the Marseille coastal area for the two versions of the biogeochemical model, with and without the P cycle.

An evaluation of a 3D ecosystem model starts with the hydrodynamics. Pairaud et al. [6] performed the hydrodynamics study for the years 2007 and 2008. They computed model/observation statistics and showed the ability of the model to capture monthly to seasonal variability in the thermal structure and to reproduce the observed features over the shelf, such as the warming or cooling of the sea due to upwelling and downwelling events and the punctual extension of the Rhone River plume.

Realistic 3D simulations were performed using the coupled model for 2007 through 2011. First, we present the model-observation comparisons, where we examined the temporal dynamics in the surface and bottom layers at the Somlit station and computed statistics. Then, we present spatial maps to assess the representation of the spatial gradient and their variability during the year. Finally, we focused on short time scale shelf processes and their impacts on the biogeochemical functioning of the pelagic coastal ecosystem.

### 1. Observations/model comparisons at the Somlit station

#### 1.1. Temporal dynamics

**Figure 3:**
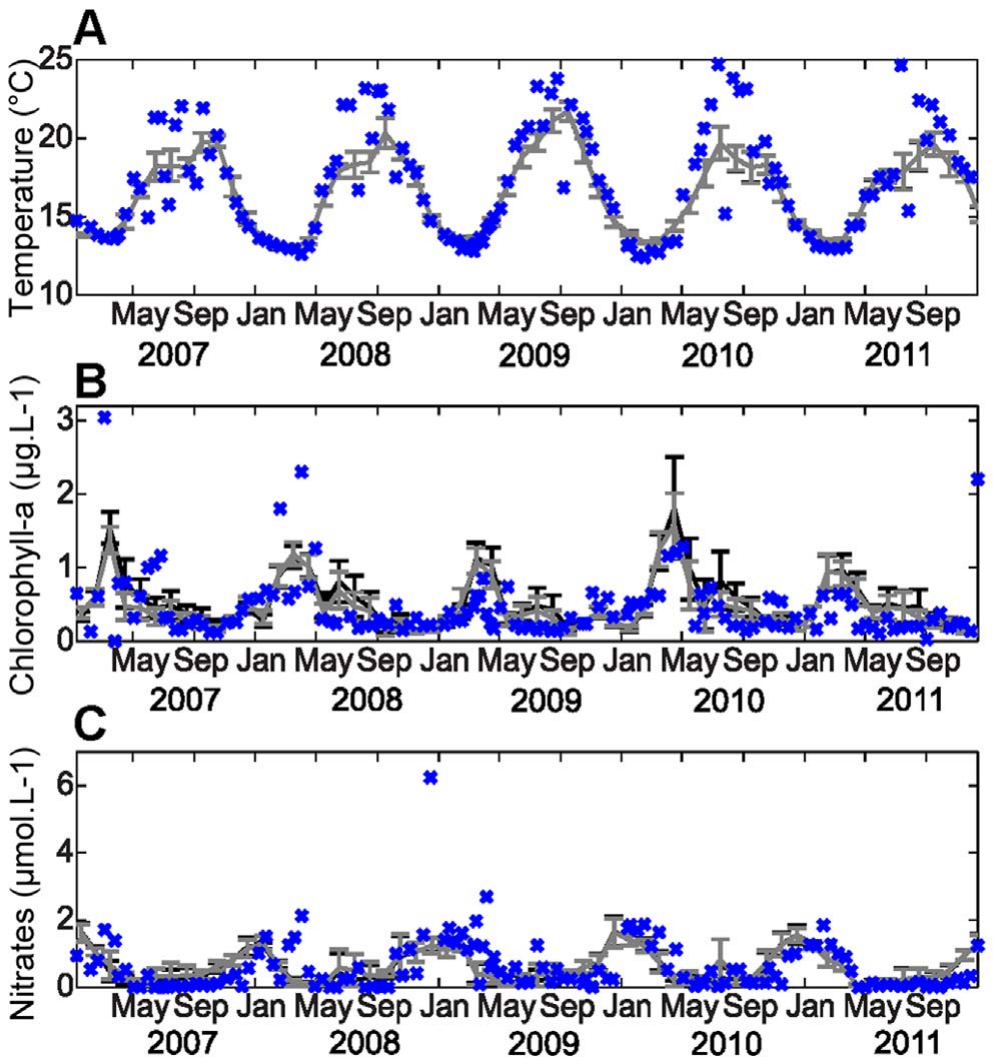
Surface model/data time series comparisons for temperature, chlorophyll-a and NO_3_. Time series of the monthly mean temperature (°C)(A), chlorophyll-a concentration (µg.L-1) (B) and NO_3_ concentration (µmol.L-1) (C) from the model with the P cycle (grey) and the model without P (black), with one standard deviation (error bar), compared with the in-situ data (blue) at the Somlit station.

**Figure 4:**
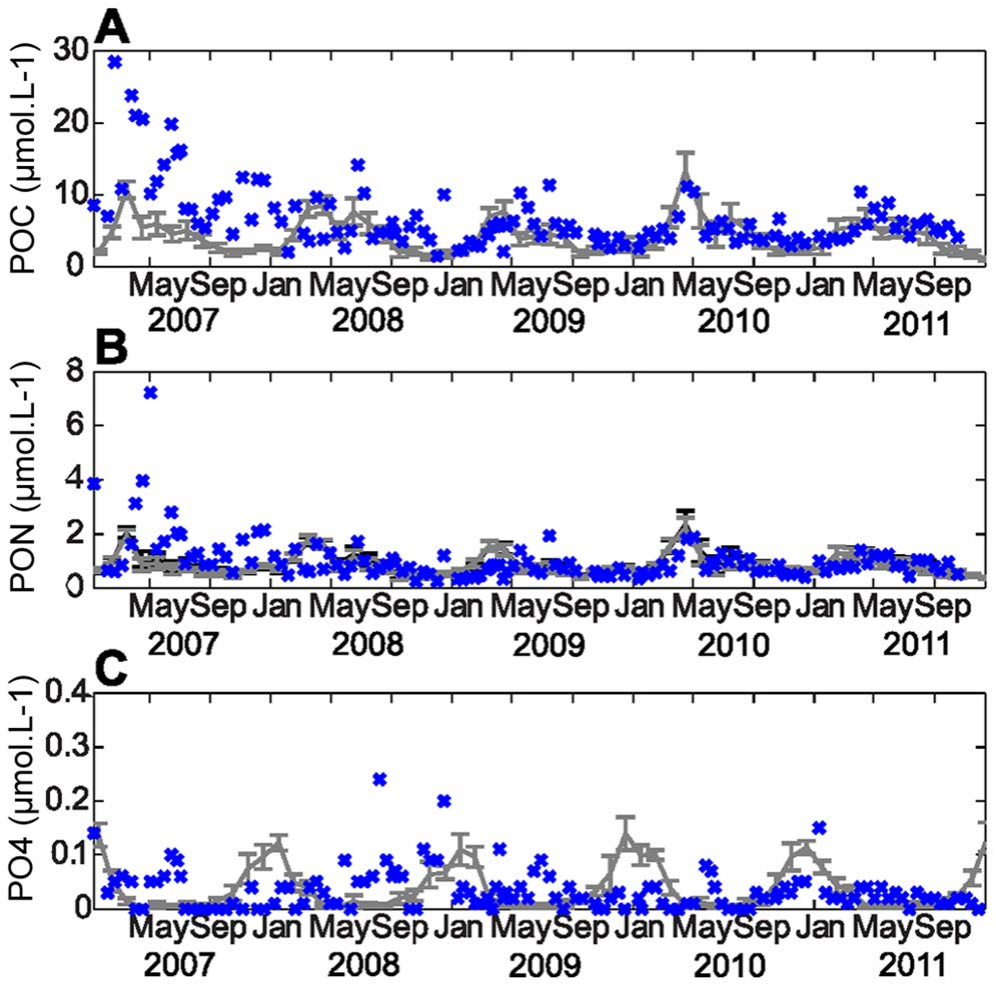
Surface model/data time series comparisons for POC, PON and PO_4_. Time series of the simulated monthly mean POC (µmol.L-1) (A), PON (µmol.L-1) (B) and PO_4_ (µmol.L-1) (C) concentrations from the model with the P cycle (grey) and the model without P (black), with one standard deviation (error bar), compared with the in-situ data (blue) at the Somlit station.

To estimate the reliability of the model in qualitative terms, we described the agreement between simulated and observed monthly means, in-situ measurements of temperature, chlorophyll-a, NO_3_, particulate organic matter and PO_4_ (Figure 3–4). At the Somlit coastal station (Annex S4), both model versions (with and without the P cycle) captured the annual cycle, with strong seasonal oscillations in temperature, NO_3_ and chlorophyll-a. The error bars showed the variability at a smaller time scale than the month of the simulation. If the winter temperatures were estimated well, the maximum summer temperatures seemed to be significantly underestimated. Nitrate concentrations and seasonal evolution were well quantified by the model. Higher chlorophyll-a variability was observed over shorter time scales in spring and summer than in winter and autumn (Figure 3). Both versions of the model reproduced the winter period well when the vertical mixing induced high NO_3_ concentrations and low temperature in the surface layer. The spring was characterized by high chlorophyll-a concentrations (spring bloom) associated with a decrease in NO_3_. Peaks in the chlorophyll-a concentrations during this season were slightly overestimated by both model versions (Figure 3). During summer, nutrient depletion was reproduced well, while the chlorophyll-a concentration was overestimated. Some isolated values of nitrate and chlorophyll-a were captured by the model.

Both versions of the model demonstrated their abilities to capture inter-annual variability, with a higher spring peak in chlorophyll-a (Figure 3), POC and PON (Figure 4) in 2010 than in 2011. In 2009, the chlorophyll-a concentration increased in November and December, which was not the case for 2007, and the model reproduced this inter-annual variability well.

The model suggests a clear and regular seasonal variation for phosphate, similar to those for nitrate, which is not the pattern observed in the field by bi-weekly sampling. The concentrations in PO_4_ remained very low (lower than 0.2 µmol.L^−1^) throughout the year, which made it difficult to distinguish a seasonal signal in the in-situ PO_4_ data. The two model versions had rather similar results over the five years. However, there were a few discrepancies between them in chlorophyll-a, POC and PON, particularly in summer and spring when the model version without the P cycle overestimated the chlorophyll-a concentration more than the model version with the P cycle (Figure 3, Figure 4). Thus, the addition of the P cycle improved the results during spring and summer.

#### 1.2. Statistical model/observations comparisons

This qualitative comparison was completed through the calculation of statistical indicators between the Somlit time series and the results of the simulations (only for the years 2009 to 2011). The surface and bottom results comparisons are presented in Table 3 and Table 4, respectively. Although the Somlit station is a coastal station near Marseille, the low mean concentrations of NO_3_, chlorophyll-a and PO_4_ (both in model and in observations) indicated that oligotrophic conditions were predominant. The standard deviation of the in-situ data was frequently equal to or very close to the mean concentration at the surface for NO_3_ (0.68±0.64 µmol.L-1), PO_4_ (0.03±0.03 µmol.L-1) and chlorophyll-a (0.4±0.32 µg.L-1), which highlights that this oligotrophic ecosystem had a strong variability, possibly due to pulsed nutrients. This variability made the model/observations comparisons difficult, so that different statistical indicators were used.

**Table 3:** Surface model and data comparison at the Somlit station from 2009 to 2011.

	TEMP	SAL	CHL		NO_3_		NH_4_		O_2_		POC		PON		PO4
			P	No P	P	No P	P	No P	P	No P	P	No P	P	No P	P
n	78	78	78	78	73	73	77	77	74	74	73	73	73	73	64
Mean (in situ)	17.15	37.97	0.40	0.40	0.68	0.68	0.31	0.31	241.12	241.12	5.13	5.13	0.79	0.79	0.03
Mean (model)	16.59	37.99	0.52	0.58	0.58	0.56	0.13	0.13	231.55	233.46	3.59	4.29	0.88	0.93	0.04
Std (in situ)	3.63	0.17	0.32	0.32	0.64	0.64	0.68	0.68	14.19	14.19	2.09	2.09	0.35	0.35	0.03
Std (model)	2.68	0.36	0.52	0.64	0.57	0.59	0.20	0.26	18.66	20.89	2.85	3.71	0.59	0.67	0.05
CF	0.27	1.24	1.01	1.16	0.80	0.81	0.40	0.43	1.30	1.37	1.20	1.19	1.17	1.19	1.39
Bias (%)	3.21	−0.05	−28.75	−44.41	14.17	17.05	59.95	59.65	3.97	3.18	30.03	16.30	−10.96	−16.84	−15.35
AAE	0.99	0.21	0.33	0.38	0.51	0.51	0.27	0.29	18.39	19.47	2.49	2.48	0.41	0.41	0.04
RMSD	1.37	0.33	0.52	0.63	0.7	0.7	0.73	0.75	22.56	23.94	3.17	3.38	0.55	0.6	0.05
R	0.96	0.44	0.34	0.34	0.34	0.36	0.00	−0.01	0.24	0.20	0.40	0.47	0.41	0.46	0.10

n: the number of in-situ data available for comparison, std : the standard deviation.

**Table 4:** Bottom model and data comparison at the Somlit station from 2009 to 2011.

	TEMP	SAL	CHL		NO_3_		NH_4_		O_2_		POC		PON		PO4
			P	No P	P	No P	P	No P	P	No P	P	No P	P	No P	P
n	74	74	74	74	71	71	73	73	67	67	69	69	69	69	63
Mean (in situ)	14.56	38.04	0.36	0.36	1.03	1.03	0.14	0.14	243.53	243.53	4.16	4.16	0.66	0.66	0.03
Mean (model)	14.78	38.16	0.81	0.77	0.5	0.52	0.11	0.11	233.38	233.5	6.03	6.3	1.5	1.47	0.09
Std (in situ)	1.75	0.09	0.22	0.22	0.89	0.89	0.11	0.11	12.38	12.38	1.6	1.6	0.28	0.28	0.03
Std (model)	1.25	0.11	0.56	0.52	0.58	0.58	0.15	0.15	17.41	17.92	3.3	3.55	0.66	0.65	0.06
CF	0.42	1.75	2.39	2.23	0.88	0.87	1.08	1.08	1.27	1.29	1.89	2.03	3.21	3.09	2.32
Bias (%)	−1.55	−0.32	−122.52	−111.72	51.96	50.23	20.15	16.66	4.17	4.12	−44.98	−51.49	−128.24	−123.06	−164.91
AAE	0.74	0.15	0.52	0.49	0.78	0.77	0.12	0.12	15.68	15.94	3.02	3.25	0.89	0.85	0.07
RMSD	0.95	0.17	0.71	0.65	1.09	1.08	0.18	0.18	19.49	19.76	3.84	4.15	1.06	1.03	0.08
R	0.86	0.29	0.25	0.26	0.21	0.22	0.06	0.05	0.41	0.41	0.19	0.2	0.24	0.26	0.04

First, we computed the cost function. According to the OSPAR classification [76], our results were very good or good except for bottom chlorophyll-a, POC, PON and PO_4_. Then, we computed the average absolute error (AAE, Equation 10) and the RMSD, which measure the average magnitude of the errors in a dataset, without considering their direction. AAE and RMSD were expressed in the same units as the variable. In the surface layer, the addition of the P cycle induced a decrease or stagnation in the AAE for all of the variables, except for the POC, for which the increase in the AAE was not significant. The model version with the P cycle performed better at representing the chlorophyll-a concentration, with an AAE of 0.33 µmol.L^−1^ versus 0.38 µmol.L^−1^ for the model version without the P cycle. In the bottom layer, the AAEs were higher than in the surface layer, except for the NH_4_, salinity and O_2_. The addition of the P cycle induced slight variations or stagnations in the AAE for all the variables. The model with the P cycle performed slightly worse for the bottom chlorophyll-a concentrations, with an AAE of 0.52 versus 0.49 µmol.L^−1^ for the model without the P cycle.

**Figure 5:**
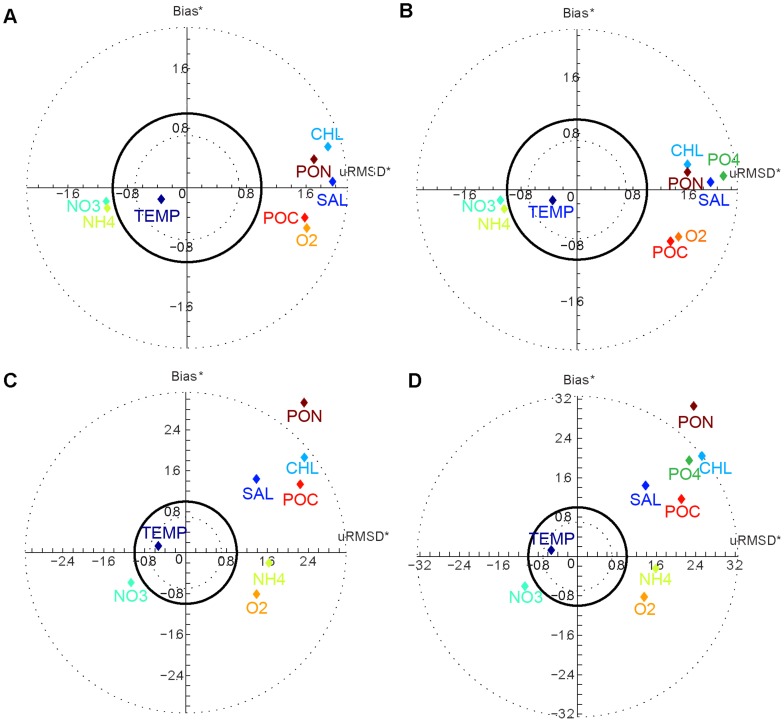
Target diagrams of the model/data comparison. Target diagrams of a set of variables for the model with the P cycle (A–C) and without the P cycle (B–D). The model/data comparison is made using in-situ data for the years 2009 to 2011 at the surface (A–B) and at 55 m (bottom C–D) at the Somlit station. The normalized bias (dimensionless) was computed versus the sign unbiased normalized RMSD (dimensionless).

Target diagrams [78] provide summary and visual information regarding the bias and the error between a model and observations. In order to compare variables with each other normalized statistics were used as described in Jolliff et al. [78]. In the Target diagram presented (Figure 5), the Y-axis corresponds to the normalized bias (Bias*) and the X-axis corresponds to the normalized unbiased RMSD (uRMSD*). Variables located in the upper part of the diagram (Y>0) were overestimated by the model (chlorophyll-a, PON and PO_4_). For the X-axis, the model standard deviation is larger than the standard deviation for the observations when variables are located in the right part of the target (X>0). The distance between any point and the origin is then the value of the total RMSD (see Jolliff et al. [78] for more details). As shown by the time series and previous comparisons, the discrepancies between the two versions of the biogeochemical model are small both at the surface (Figure 5 A–B) and at the bottom (Figure 5 C–D). The main differences were in the chlorophyll-a concentrations, for which the model version with the P cycle was more efficient in the surface layer with a lower normalized bias and a smaller uRMSD* than the model version without the P cycle. Both model versions overestimated the amplitude of the variations in the state variables except for TEMP, NO_3_ and NH_4_. The target diagrams also demonstrate that models were less efficient in representing the bottom layer, with an increased bias for PON and POC.

To summarize, the timing and magnitude of the surface chlorophyll-a and NO_3_ concentrations were generally well matched. The shortcomings of the simulation were an overestimation of the chlorophyll-a concentrations in spring and a slight overestimation in summer. The model also had more difficulty in simulating the bottom than the surface at the Somlit station. However, using the in-situ data to evaluate the ability of the model as discussed above refers to data from a single station sampled every fortnight and with undoubted local bias and particularity. We thus used remote sensing data for the validation of the representation of spatial processes.

### 2. Evaluation of spatial processes representation

Ocean color observations derived from remote sensing data were used to assess the abilities of the model to capture spatial gradients and seasonality over the whole study area.

#### 2.1. Climatology of surface chlorophyll-a patterns

**Figure 6:**
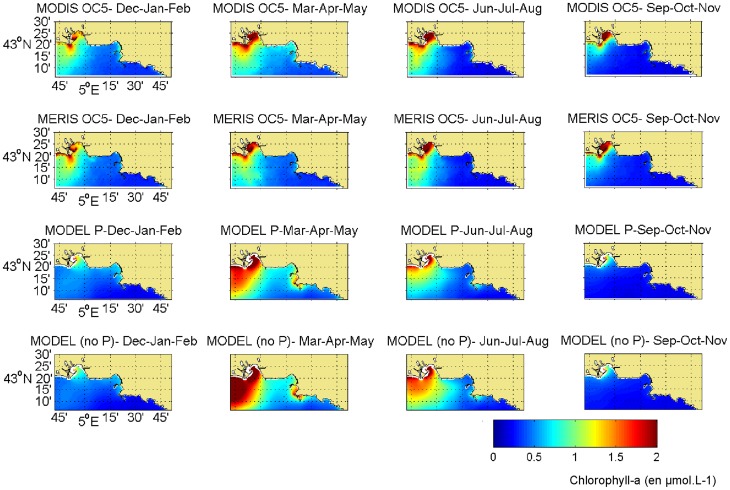
Mean surface chlorophyll concentration for winter, spring, summer and fall. Mean surface chlorophyll concentration for winter (December–January–February), spring (March–April–May), summer (June–July–August) and fall (September–October–November) for the years 2007 to 2011 from the remote sensing data (MODIS and MERIS) and the simulation (with and without the P cycle). The model results presented were averaged across the 10 first meters of the water column.

The maps of time-averaged surface chlorophyll-a concentrations permit the evaluation of the ability of the model to reproduce horizontal patterns of chlorophyll-a. The outputs from both versions of the model were compared with observed maps of surface chlorophyll-a concentrations from MODIS and MERIS during the 4 seasons of the year (Figure 6). The Rhone River plume area was characterized by high chlorophyll-a concentrations, in contrast with the eastern portion of the study area, where concentrations in chlorophyll-a remained very low. The models captured the west-east gradient in surface chlorophyll-a concentration. The BoM seems to be a transition zone between the rich water of the Rhone River plume and the oligotrophic water located eastward. The models reproduced well the spatial extension of the Rhone River intrusion plume, except very close to the mouth, where the concentrations were underestimated by the model. This underestimation was caused by the inability to distinguish terrestrial and fluvial chlorophyll-a from marine chlorophyll-a in the remote sensing data. The marine chlorophyll-a is the only one included in the model.

The chlorophyll-a remote sensing data were highly variable in both the spatial distribution and the concentrations; this variability was well captured by both versions of the model. In winter, both version of the model reproduced well the chlorophyll-a concentrations in the BoM, but the concentrations associated with the Rhone River plume were underestimated. During spring and summer, the model fields deviate from the observations in overestimating the remotely sensed chlorophyll-a concentrations in the Rhone River plume. Nevertheless, the spring remains the season during which the surface layer was the most productive and the chlorophyll-a concentration was at its maximum in the BoM. The summer period had lower concentrations than in the spring in the surface layer, particularly in the BoM. The fall was characterized by the lowest chlorophyll-a concentrations of the year in the entire study area; the signal of the Rhone River was weak in comparison with the three other seasons.

The spatial comparisons exhibited little discrepancy between the two versions of the model. During spring and summer, the chlorophyll-a signal associated with the Rhone River plume was lower and more similar to the observations in the model with the P cycle both spatially and in intensity. Therefore, the addition of the P cycle causes an improvement in the representation of the effects of the Rhone River on chlorophyll-a concentrations.

### 3. Influence of short time scale shelf processes forcing on co-limitation functioning

**Figure 7:**
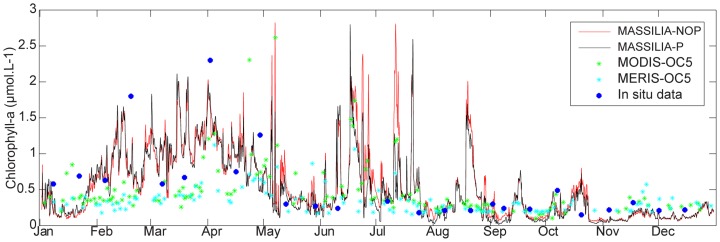
Surface chlorophyll concentrations for the year 2008. Evolution of the surface chlorophyll concentrations from the remote sensing data of MODIS (green point) and MERIS (blue point), in-situ data (blue point) and the simulation with the P cycle (black line) and without the P cycle (blue line) at the Somlit station.

In the evaluation of the representation of short time scale shelf processes in the coupled models, we focused on the year 2008, for which hydrodynamic shelf processes off Marseille were analyzed in detail by Pairaud et al. [6]. The Marseille coastal area was under strong forcing influences (upwelling events, intrusion of Rhone River plume in the BoM), which induced significant daily variability in the biogeochemical variables. Figure 7 presents the observations and daily model results for 2008 used to evaluate the ability of the model to reproduce processes over short time scales. In early April, a peak in chlorophyll-a was present in the model results and in the remote sensing and in-situ data, and we noticed strong discrepancies between the in-situ data and the remote sensing data. The model values were between those of the remote sensing and in-situ data, leading to the assumption that chlorophyll-a concentrations could be underestimated from remote sensing data in spring. In summer, the BoM experienced enrichment events, when modeled chlorophyll-a concentrations increased from 0.5 to 1.5 µmol.L^−1^. Those events matched the observations very well, except for the event of late August 2008. The chlorophyll-a concentrations remained low during fall with slight variations in intensity for both the models and observations. Weak discrepancies were observed at short time scales between the two versions of the model, except in summer, when enrichment events were clearly less intense in the model with the P cycle. To explain these discrepancies, we focused on phytoplankton limitation by P and N.

**Figure 8:**
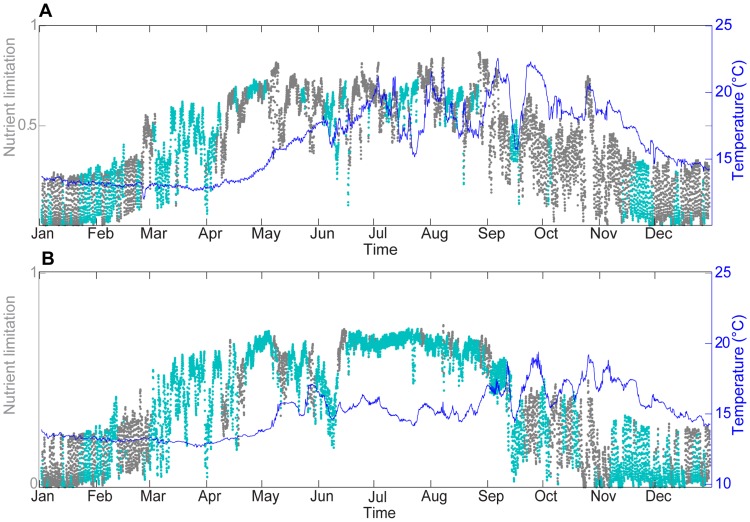
Evolution of temperature and phytoplankton nutrient limitation for the year 2008. Phytoplankton limitation by P (grey point) and N (blue point) and model temperature (blue line) for the year 2008 at the Somlit station at the surface (A) and at 60 m (B).

Figure 8 presents phytoplankton limitation by nutrients calculated using the cell quota formulation in the surface and bottom layers at the Somlit station. As the limitation increases in value toward 1, the nutrient limitation on phytoplankton increases. Temperature was also presented on the same graph (Figure 8) to evaluate the links between nutrient co-limitation and temperature. The results of the model showed that phytoplankton limitation by nutrients was greater in the surface layer (Figure 8A) than in the bottom layer (Figure 8B), as was the variability in limitation. As shown in Figure 3, high concentrations of nutrients were available from November to March due to winter vertical mixing, so that phytoplankton limitation by nutrients remained very low in the surface and bottom layers. An increase in limitation occurred from March to the beginning of May, corresponding to the spring bloom. The end of the spring bloom was marked by the exhaustion of nutrient supplies in the surface layer, the stabilization of limitation at high levels and the stabilization of the chlorophyll-a concentration at approximately 0.3 µg.L^−1^ at the surface (Figure 7). Short, strong decreases in limitation happened during summer at the surface. Then, the fall was associated with a decrease in limitation by nutrients. Therefore, phytoplankton limitation by nutrients had an apparent seasonal signal, and increases in the chlorophyll-a concentration in the surface layer were linked with short decreases in phytoplankton nutrient limitation. However, phytoplankton nutrient limitation was not directly correlated with chlorophyll-a because temperature and light limitation were also considered to affect chlorophyll-a seasonality.

The model results also showed that phytoplankton were more limited by P (grey points) in the surface layer and more by N (green points) in the bottom layer. However, switches between P and N limitation occurred several times during the year, highlighting the strong variability of ecosystem functioning most likely due to physical shelf processes.

#### 3.1. Influence of upwelling

The Marseille coastal area is strongly impacted by upwelling events associated with strong upwards vertical velocity (maximum of 5 cm.s^−1^ during an upwelling event in November 2008), which led to an important decrease in surface temperature [6]. Significant upwelling events occurred during the summer of 2008, and these strong temperature decreases were always associated with N limitation (Figure 8A). This result was consistent with the modeled N limitation of phytoplankton development in bottom layer (Figure 8B).

**Figure 9:**
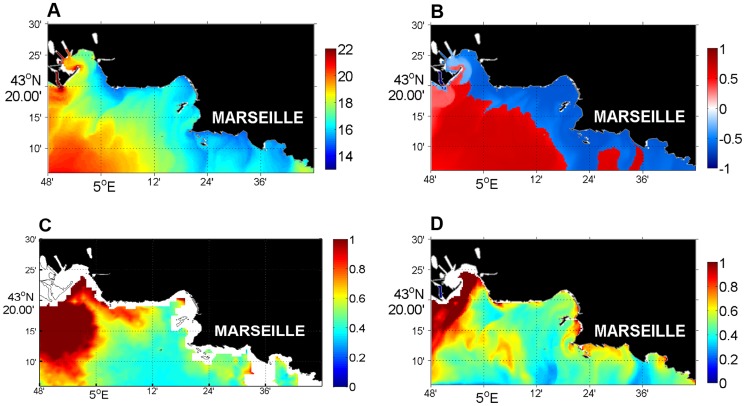
Upwelling event of 15/07/2008. Simulated temperature (A), simulated phytoplankton limitation by P (red) and N (blue) (B), chlorophyll-a concentrations from MODIS and simulated chlorophyll-a concentration (average across the first 10 meters of depth) compared for 15/07/2008.

The maps in Figure 9 present a comparison of surface temperature, chlorophyll-a concentration and phytoplankton nutrient limitation during an upwelling event in July 2008. During this event, the area impacted by the ascent of deep water was characterized by low temperature (T<20°C) (Figure 9A) and limitation by N rather than P (Figure 9B). The lower temperature was localized to the two primary upwelling points (“Cote Bleue” and “Calanques”), but the majority of the Marseille coastal area was impacted by upwelling as described by Pairaud et al. [6]. The remote sensing data and modeled chlorophyll-a concentrations were in good agreement (Figure 9 C–D); the concentrations were high in the two primary upwelling points in response to the nutrient enrichment induced by the upwelling. A weak increase in chlorophyll-a was observed far from the two main upwelling spots but still located in the temperature-impacted upwelling area. This could be explained by the rapid uptake of nutrients near the upwelling points. The nutrients were consumed by phytoplankton before being transported away from the upwelling points due to the strong nutrient limitation of phytoplankton in the summer and in the surface layer. These results explained why the surface temperature was strongly impacted and the surface chlorophyll-a concentration more weakly impacted at the Somlit station during upwelling events (Figure 7–8A).

#### 3.2. Influence of the intrusion of water from the Rhone River in the BoM

**Figure 10:**
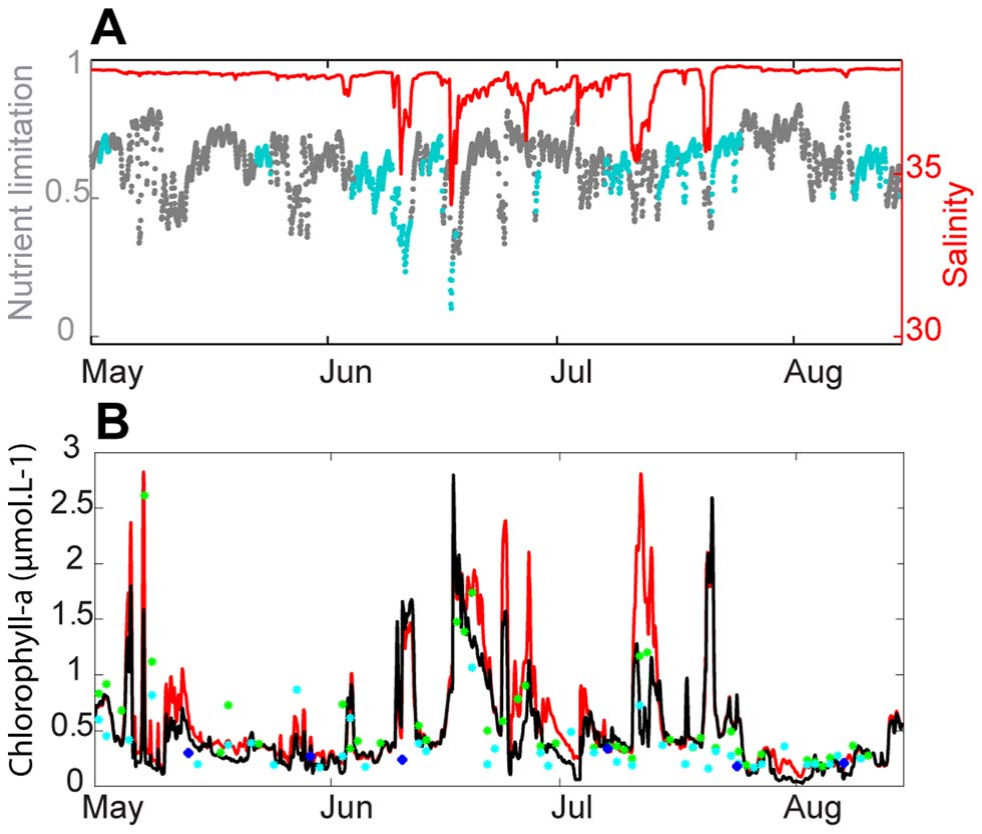
Impact of the intrusion of Rhone River water on phytoplankton nutrient limitation and chlorophyll-a concentrations. Evolution from May to mid-August 2008 at the Somlit station of the simulated phytoplankton limitation by P (grey point) and N (blue point) and the simulated salinity (red line) (A), the surface chlorophyll-a concentrations from remote sensing observations of MODIS (green point) and MERIS (blue point), the in-situ data (bleu point) and the simulation with the P cycle (black line) and without the P cycle (blue line) (B).

The intrusion event of water from the Rhone River into the BoM that was studied by Pairaud et al. [6] occurred in the second part of June 2008 and was detected in the model results at the Somlit station as a decrease in salinity (Figure 10A). The study of the summer decreases in phytoplankton nutrient limitation revealed that they were linked to low salinity events. Rich Rhone River water induced an increase in nutrients and thus a decrease in phytoplankton nutrient limitation (Figure 10A). Both versions of the model were in agreement with the observed chlorophyll-a concentrations (Figure 10B), highlighting the strong biological response to the Rhone River water intrusion events. The intensity of the increases in chlorophyll-a were lower in the version of the model with the P cycle during most of the low salinity events. Therefore, according to the results of the model, phytoplankton was limited by P during most of the low salinity events. This finding was in agreement with spatial comparisons of chlorophyll-a concentrations in the Rhone River plume (Figure 6), which were less extended and better represented in the model version with the P cycle. Therefore, the P cycle slightly improved the results, such as during the low salinity event in mid-July where a net improvement was noticed.

## Discussion

Due to a worldwide movement toward ecosystem-based management [79], the demand for quantitative tools to support ecosystem-based management initiatives increases [79], [80]. There is now a proliferation of end-to-end ecosystem models which attempt to represent the entire ecological system and the associated abiotic environment [81]. This kind of models has been developed in various areas [82], [83] and some are listed in the MEECE European project (http://www.meece.eu). However this kind of models has lots of parameters, needs a lot of input data, human and material resources to be developed, which make them difficult to transpose in numerous areas.

In this study, we preferred a different modeling approach and we developed a model adapted to targeted questions: study of the impact of the physical processes on low trophic levels and study of the inputs of chemical contaminants in the trophic chain. So, we chose to develop a physical biogeochemical coupled model with a high spatial and temporal resolution and with an on-line coupling technique in order to describe finely the link between physics and biogeochemistry. This coupled model focused on the low trophic level in order to study the key processes which permit the input of contaminant in the trophic chain. The biogeochemical model with a low number of parameters relatively to end-to-end models is easier to adapt to other oligotrophic coastal area influenced by large river inputs like the Gulf of Gabes in Tunisia and the Nile delta in eastern Mediterranean Sea. Indeed, the biogeochemical model ECO3M-MASSILIA-P was modified from an initial model [40], [41], [84].

During the biogeochemical model development, the following question of the complexity level of the biogeochemical model was asked: What was the level of detail necessary and sufficient to describe this coastal ecosystem? The Mediterranean Sea is generally known to be P limited; thus, we decided to add the P cycle to the model. In choosing the most reliable model, particular attention was paid to the accuracy of the model and the assessment of the shortcomings of the two new versions of the biogeochemical model.

### 1. Model accuracy assessment

The models simulated 5 years, from 2007 to 2011. The model development and parameterizations were performed for 2007 and 2008, whereas model accuracy assessment of both final model versions was performed for 2009 to 2011, which allowed a robust evaluation of the model results.

The results showed that both versions of the model had significant ability to simulate the biogeochemical functioning of the Marseille coastal area. Indeed, the models reproduced with relatively good accuracy the seasonal and the inter-annual dynamics of chlorophyll-a and nutrient concentrations. Nevertheless, the chlorophyll-a concentration was slightly overestimated by the model during the spring bloom and in summer. The models reproduced the spatial pattern of this area well, with a good representation of the west-east gradient in chlorophyll-a concentration associated with the Rhone River plume extensions. The general ability assessment of model results also showed that there was strong variability in biogeochemical concentrations at short time scales (from a few hours to several days), mostly in spring and summer. The model was able to reproduce different types of shelf events, such as upwelling or the intrusion of Rhone River water into the BoM, and their associated biogeochemical response. The use of target diagrams allowed a visual comparison of statistical indicators. The results appeared more accurate at the surface than at the bottom, and the discrepancies between the two versions of the biogeochemical model were rare.

Nevertheless, the implementation of the P cycle in the biogeochemical model improved the agreement between the model predictions and the surface chlorophyll-a observations for a given event. In addition, the model with the P cycle performed well at reproducing the plume and processes associated with the Rhone River. However, in the bottom layer, its use increased the error in predicting the chlorophyll-a concentration due to the overestimation of the PO_4_ concentration. We attributed the improved fit in the surface chlorophyll-a concentrations to the ability of the model to reproduce the P limitation in the spring and summer periods. In winter, phytoplankton production was principally limited by light because the strong vertical mixing brought nutrients in excess to the surface layer. During this period, the P cycle did not impact the surface chlorophyll-a concentration.

### 2. Model complexity

The general assessment of the results of both versions of the model showed that the accuracies of the models with and without the P cycle were similar. The addition of the P cycle to the biogeochemical model improved the description of the ecosystem functioning, but it also injected errors.

The errors associated with the P cycle came from different sources. The primary difficulty was that the measured PO_4_ concentrations in the Marseille coastal area were often lower or very close to the detection limit. At the Somlit station in the surface layer, the concentration of PO_4_ ranged from 0 to 0.3 µmol.L^−1^, with a mean value of 0.03 µmol.L^−1^ for the years 2009 to 2011, which indicated that concentrations of PO_4_ were generally lower than or close to their detection limits, as the precision of the PO_4_ concentrations was ±0.02–0.03 µmol.L^−1^. Therefore, comparisons between the model and the observations were not possible. Then, as previously stated by several authors [85]–[87], there is a critical need for new experimental data examining N and P dynamics under different and extreme input ratios and growth rates [48]. Thus, the need for improved biogeochemical understanding of N/P co-limitation increased the difficulty of modeling the P cycle. Furthermore, Poggiale et al. [88] demonstrated that models were sensitive to uptake formulations; even if two formulas provided similar values, large numerical differences in the stability criteria may occur. Thus, errors were certainly introduced by the choice of the parameter values and the mathematical functions representing the P cycle.

Even if it appears plausible from a biological a priori standpoint that more complex models should mirror reality better, other recent studies have suggested that it is not always the case. A meta-analysis of mechanistic aquatic biogeochemical models also found that increased model complexity did not improve the fit [37]. Kriest et al. [89] suggested that increasing complexity of unturned, unoptimized models that were simulated with parameters commonly used in large-scale model studies did not necessarily improve performance. Los et al. [90] also indicated that adding more complexity did not necessarily improve the quality of the model results in terms of their ability to reproduce measurements and hence their applicability as prognostic tools. Instead, they argued that there should be a balance between ecological and physical resolution in relation to the specific question to be addressed. Crout et al. [91] evaluated models using model reduction for three models. In all cases, they identified reduced models that outperformed the more complicated ones, “suggesting some over-parameterization has occurred during model development” [91].

In the study of the Marseille coastal area, we followed an inverse approach, with the simplest model (model without P cycle) at the beginning and consecutively increasing model complexity with the P cycle. The addition of the P cycle increased the computation time by approximately 30% and did not automatically improve the fit with the observations. Therefore, the model version (with or without the P cycle) should be chosen depending on the question to be addressed and by considering the general accuracy assessment achieved in this work.

### 3. The Marseille Coastal area: a challenging zone for a modeling approach

The region of interest is very complex (biologically and dynamically) and was thus a challenge to model. Principally, 4 difficulties were encountered: (i) there was a strong west-east gradient in oligotrophy, namely the eastern portion of the studied area was mainly oligotrophic, while the western portion was more productive; (ii) few observations were available for comparisons with the model results; (iii) the studied region was a coastal area highly influenced by large river inputs (the Rhone River and the Marseille urban rivers); and (iv) the hydrodynamic shelf processes were extremely variable at short time scales, and they strongly impacted most of the coastal domain.

The BoM was generally oligotrophic and characterized by low concentrations of chlorophyll-a and nutrients, with mean concentrations of chlorophyll-a, PO_4_ and DIN of 0.4 µg.L^−1^, 0.03 µmol.L^−1^ and 1 µmol.L^−1^, respectively. In comparison, in the surface layer of the Baltic Sea, the mean concentrations of chlorophyll-a, PO_4_ and DIN were approximately 3.5 µg.L^−1^, 0.33 µmol.L^−1^ and 3.6 µmol.L^−1^ [92]. As in the Baltic Sea, a modeling study in the Channel and Southern Bight of the North Sea showed mean concentrations of chlorophyll-a of 5.35 µg.L^−1^, mean PO_4_ of 0.82 µmol.L^−1^ and mean DIN of 17.57 µmol.L^−1^ [77]. Therefore, in the BoM, the concentrations were ten times less than the concentration range in the Channel and Southern Bight of the North Sea and in the Baltic Sea. The BoM contrasted with the Rhone River plume, which was characterized by high concentrations of nutrients and chlorophyll-a. Thus, the large range of trophic conditions in this small model domain put the model to the test.

The scarcity of observations to compare with the model results was a problem during the model development phase. The Somlit station was sampled only twice monthly, and as shown by the model results, many processes occurred at shorter time scales. One risk to avoid was to tune the model to obtain good results in comparison with the in-situ data at the Somlit station. We also evaluated the spatial pattern of the processes reproduced by the model and compared them with remote sensing data, which provided spatial patterns and gave daily information (during non-cloudy conditions). Nevertheless, the errors associated with the remote sensing data were high.

Coastal areas are highly influenced by cross-shore and earth/sea exchange. The Marseille coastal area has two large open boundary conditions (OBC). Thus, a good representation of the OBC was essential to accurately describe the cross-shore exchanges. Coastal modeling also requires long-term monitoring of inputs to represent the discharge and concentrations associated with each river well. Although the Rhone River was well-studied and the daily discharge and concentrations of the primary state variables were available, it was necessary to construct from the literature or from the few available observations the concentrations for the missing variables for the Marseille urban rivers and the Rhone River. Progress could be made in the description of the concentrations of the rivers by monitoring each river to better describe the organic matter and nutrient inputs from the rivers.

In addition, the low impact of the addition of the P cycle to the model suggested that physics drives the majority of the coupled model results. Hydrodynamic shelf processes appeared extremely variable at short time scales, and they strongly impacted most of the coastal domain. In coastal areas, hydrodynamics were of primary importance for coupled modeling. An overestimation of the plume extension of the Rhone River by the hydrodynamic model induced a strong error in the chlorophyll-a concentration at the Somlit station, which demonstrated that the choice of the physical model is very important for biogeochemistry, as stated in many studies [93], [94].

### 4. Contribution of the model results to the understanding of the Marseille coastal area

As previously discussed, although this coastal ecosystem was complex and thus a challenge to model, the coupled model performed well at reproducing the primary characteristics and processes. Therefore, the coupled model provided interesting information on the functioning of this coastal ecosystem.

First, the temporal evolution of surface chlorophyll-a clearly distinguished the four main seasons. Spring was characterized by high concentrations of chlorophyll-a (spring bloom) and was the most productive season. In contrast, the fall appeared to be the least productive period. This finding is not in accordance with general knowledge of the Northwestern Mediterranean Sea as a small autumnal bloom is supposed to occur during this period. Our coupled model reproduced the autumnal bloom only for 2009.

From a spatial point of view, the Marseille coastal area was characterized by a strong west-east oligotrophy gradient. Outside the ROFI, the coastal domain near Marseille generally remained oligotrophic, whereas at the mouth of the Rhone River, the phytoplankton biomass was always high [95]. Nevertheless, the Rhone River was limited by P because the NO_3_∶PO_4_ ratio of the Rhone River inputs was approximately 65–80 [26], [27]. This P limitation was also detectable in the model results because the chlorophyll-a concentrations in the Rhone River plume were higher in the model without the P cycle than in the model with the P cycle, better mimicking the observations.

The urban rivers and the Marseille WWTP delivered high concentrations of nutrients and organic matter, but the eutrophication risk remained extremely low in the BoM because the Urban River inputs and sewage from the WWTP were rapidly diluted by strong hydrodynamic events [6] due to the short residence time. Nevertheless, the Gulf of Fos was characterized by shallow depths and higher chlorophyll-a concentrations (up to 10 µmol.L^−1^ [34]), which indicated that the eutrophication risk was higher. Previous studies [96] demonstrated that the greatest risk of algal blooms occurs during periods of calm weather because the Gulf of Fos is flushed intermittently but strongly by wind-driven lateral circulation [34].

The coupled model permitted the evaluation of the eutrophication risk in the BoM, but the model was also very useful in contextualizing the in-situ data. Indeed, the Somlit station is impacted by many shelf events (e.g., upwelling and the intrusion of Rhone River water). For example, an increase in nutrient and chlorophyll-a concentrations associated with a decrease in salinity could be caused by the intrusion of Rhone River water, heavy rainfall events or an extension of the urban river plumes.

The coupled model presented a significant advantage over satellite images and in-situ measurements by allowing the study of the processes at short time scales and in three dimensions. It was proven to be a pertinent tool in the study of the biological response associated with physical processes. The study of upwelling events in summer 2008 showed that a large part of the Marseille coastal area was impacted by upwelling events. However, the chlorophyll-a response to the nutrient enrichment caused by upwelling was mostly noticeable at the two main upwelling points (“Cote Bleue” and “Calanques”). Another interesting event was the intrusion of Rhone River water into the BoM. The influence of the Rhone River water in the BoM has been observed hydrodynamically [6], [21], but the only impacts on the ecosystem that had previously been investigated were in the CDOM concentrations [22]. The coupled model revealed that the intrusion of Rhone River water into the BoM induced an obvious increase in chlorophyll-a concentrations.

Finally, further studies are necessary to better explore and characterize the biogeochemical functioning of this coastal ecosystem. The coupled model allowed the quantification of the impact of atmospheric inputs (organic matter and nutrients) on the biogeochemical functioning of this coastal ecosystem. Preliminary results (not shown) suggested that atmospheric deposits might be negligible, except during episodes of heavy rainfall. The initial results concerning the intrusion of Rhone River water also raised new questions: What caused the eastward transport of Rhone River water? What quantities of nutrients are transported into the BoM during Rhone River water intrusion events? Thus, the coupled model could be a useful tool in quantifying the relative impact of the Rhone River and the Urban Rivers on the biogeochemical functioning of the BoM using a mass balance approach.

## Conclusion

In this paper, we presented the development and the evaluation of a 3D coupled physical-biogeochemical model for the Marseille coastal area. The high-resolution coupled model demonstrated its ability to recreate realistic situations. It underlined that the biogeochemical functioning of the BoM was very complex because the physical processes and river inputs were important drivers of the biogeochemical model results. Indeed, the ecosystem of the BoM quickly switched between oligotrophic conditions and important enrichment events due to external forcing.

The P cycle was added to the biogeochemical model because this element was well known to limit biological production in the Mediterranean Sea. The addition of P improved the description of ecosystem functioning and in some cases improved the model results, but it also introduced errors into the model. In using the model results, awareness of its shortcomings and positive features is important. Therefore, the general accuracy assessment in this paper and the questions addressed should be considered in choosing the version of the biogeochemical model (with and without the P cycle).

The coupled model appeared to be a useful tool for the analysis of processes and the estimation of budgets in a very dynamic environment where it is difficult to extrapolate from discrete measurements. Thus, despite its imperfections, large quantities of information are available in the results of the coupled model. The model data could help researchers to design field campaigns by better anticipating the biogeochemical front and gradient. In addition, the model results allow better evaluation of the impact of urban inputs on the coastal area and could thus help policy managers propose solutions.

In conclusion, this study suggested that improvements in the description of hydrodynamics and terrestrial inputs should be preferred over increasing the complexity of the biogeochemical model in this coastal oligotrophic area.

## Supporting Information

Annex S1 **Equations of the biogeochemical model of the Marseille coastal area (ECO3M-MASSILIA-P).**
(DOCX)

Annex S2 **Parameters.**
(PDF)

Annex S3 **Spin up of the MARS3D-RHOMA/ECO3M-MASSILIA coupled model for the year 2008 at the Somlit station at the surface.** “Model 1” corresponding to the summer initial conditions and “Model 2” corresponding to the winter initial conditions.
(DOCX)

Annex S4 **Maps of the Marseille city inputs.**
(TIF)

Annex S5 **Quality of the remote sensing data.**
(DOCX)
