# Supplementary material for: Development of a 3D Coupled Physical-Biogeochemical Model for the Marseille Coastal Area (NW Mediterranean Sea): What Complexity Is Required in the Coastal Zone?
Source: PLoS One. 2013 Dec 4;8(12):e80012. doi: 10.1371/journal.pone.0080012 (PMC3851166; doi:10.1371/journal.pone.0080012)
Supplement: Annex S1 — Equations of the biogeochemical model of the Marseille coastal area (ECO3M-MASSILIA-P). (DOCX) [file pone.0080012.s001.docx]

**Annex S1: Equations of the biogeochemical model (ECO3M-MASSILIA-P)**

Most of the equations have been described and explained in Faure et al. (2010).

# Terms signification

| **Terms** | **Definition** | **Units** |
| --- | --- | --- |
| PAR | Photosynthetically available radiation | J.m^-2^.s^-1^ |
| k | Light extinction factor | m^-1^ |
| T | Temperature | °C |
| $Q^{*}$ | Most limiting intracellular ratio | - |
| $V_{N}^{C}$ | Phytoplancton carbon specific uptake rate of nitrogen | mmol N .(mmol C)^-1^.s^-1^ |
| $V_{P}^{C}$ | Phytoplancton carbon specific uptake rate of phosphorus | mmol P .(mmol C)^-1^.s^-1^ |
| $Q_{C}^{Chla}$ | Phytoplankton Chlorophyll-a : C ratio | mg Chla .(mmol C)^-1^ |
| BP | Bacterial production | 10^12^.cell. m^-3^.s^-1^ |
| BP_c_ | Carbon bacterial Production | mmol C.m^-3^.s^-1^ |
| BR | Bacterial respiration | mmol C.m^-3^.s^-1^ |
| $V_{C_{BA}}$ | Bacterial specific uptake of carbon | mmol C.(10^12^.cell)^-1^.s^-1^ |
| $V_{N_{BA}}$ | Bacterial specific uptake of nitrogen | mmol N.(10^12^.cell)^-1^.s^-1^ |
| $V_{P_{BA}}$ | Bacterial specific uptake of phosphorus | mmol P.(10^12^.cell)^-1^.s^-1^ |

$Q_{X}^{Y}$ represents the ratio Y:X.

# Phytoplankton

## Light

Measured solar irradiance, I0 expressed in J m2 s-1, is converted in photosynthetically available radiation (PAR) with the following equation:

| $PAR_{0}=m_{1}.m_{2}.m_{3}.I_{0}$ | Equation 1 |
| --- | --- |

The vertical light attenuation was calculated using Equation 1.

| $PAR=PAR_{0}.e^{-k.z}$ | Equation 2 |
| --- | --- |
| $k=k_{0} + A_{Chl}.Chla$ | Equation 3 |

## Phytoplankton carbon

| $\frac{\partial C_{B}}{\partial t}=(1-exu_{LDOC}).P^{C}.C_{B}-g.f(T).C_{B}-R.C_{B}$ | Equation 4 |
| --- | --- |

The growth rate P^C^ :

| $P^{C}=P_{m}^{C}*\left[1-\exp\left(\frac{-\alpha_{Chl\text{a}}.PAR.Q_{C}^{Chl\text{a}}}{P_{m}^{C}}\right)\right]$ | Equation 5 |
| --- | --- |
| $P_{m}^{C}=a.e^{b*T}.Q*$ | Equation 6 |
| $Q*=\min\left(\left(\frac{Q_{C}^{N}-\min Q_{C}^{N}}{\max Q_{C}^{N}-\min Q_{C}^{N}}\right),\left(\frac{Q_{C}^{P}-\min Q_{C}^{P}}{\max Q_{C}^{P}-\min Q_{C}^{P}}\right)\right)$ | Equation 7 |

## Phytoplankton nitrogen

| $\frac{\partial N_{B}}{\partial t}=V_{N}^{C}.C_{B}-g.f(T).N_{B}$ | Equation 8 |
| --- | --- |
| $V_{N}^{C}=V_{NH_{4}}^{C}+V_{NO_{3}}^{C}$ | Equation 9 |
| $V_{NH_{4}}^{C}=\max V_{NH_{4}}^{C}.\left[\frac{\max Q_{C}^{N}-Q_{C}^{N}}{\max Q_{C}^{N}-\min Q_{C}^{N}}\right].\left(\frac{NH_{4}}{NH_{4}+K_{NH_{4}}}\right)$ | Equation 10 |
| $V_{NO_{3}}^{C}=\max V_{NO_{3}}^{C}.\left[\frac{\max Q_{C}^{N}-Q_{C}^{N}}{\max Q_{C}^{N}-\min Q_{C}^{N}}\right].\left(\frac{NO_{3}}{NO_{3}+K_{NO_{3}}}\right)$ | Equation 11 |

## Phytoplankton phosphorus

| $\frac{\partial P_{B}}{\partial t}=V_{P}^{C}.C_{B}-g.f(T).P_{B}$ | Equation 12 |
| --- | --- |
| $V_{P}^{C}=\max V_{PO4}^{C}.\left[\frac{\max Q_{C}^{P}-Q_{C}^{P}}{\max Q_{C}^{P}-\min Q_{C}^{P}}\right].\left(\frac{PO_{4}}{PO_{4}+K_{PO_{4}}}\right)$ | Equation 13 |

## Phytoplankton chlorophyll-a.

Chlorophyll-a is computed from the following equation

| $Q_{C}^{Chl\text{a}}=Q_{C}^{N}.\left(\max Q_{N}^{Chl\text{a}}.Q*+\min Q_{N}^{Chl\text{a}}\left(1-Q*\right)\right)$ | Equation 14 |
| --- | --- |

# Heterotrophic bacteria

## Bacterial production

| The bacterial production (BP) is : $BP=\mu_{\max}^{BA}.(1-f).NBA$ | Equation 15 |
| --- | --- |
| $f=\max\left(\frac{\min Q_{C_{BA}}}{Q_{C_{BA}}},\frac{\min Q_{N_{BA}}}{Q_{N_{BA}}},\frac{\min Q_{P_{BA}}}{Q_{P_{BA}}}\right)$ | Equation 16 |

## Bacterial respiration

| $BR=\rho_{BA}^{g}.BP_{C}+ \rho_{BA}^{r}.(Q_{C_{BA}}-\min Q_{C_{BA}})\cdot NBA$ | Equation 17 |
| --- | --- |
| $BP_{C}=BP.Q_{C_{BA}}$ | Equation 18 |

## Bacterial carbon

| $\frac{\partial C_{BA}}{\partial t}=V_{C_{BA}}.NBA-BR-\frac{BP.C_{BA}}{NBA}$ | Equation 19 |
| --- | --- |
| $V_{C_{BA}}=V_{LDOC}^{BA}+V_{DPOC}^{BA}$ | Equation 20 |
| $V_{LDOC}^{BA}=(1-up\_part).\max V_{LDOC}^{BA}.\left[\frac{\max Q_{C_{BA}}-Q_{C_{BA}}}{\max Q_{C_{BA}}-\min Q_{C_{BA}}}\right].\left(\frac{LDOC}{LDOC+K_{LDOC}}\right)$ | Equation 21 |
| $V_{DPOC}^{BA}=up\_part.\max V_{LPOC}^{BA}.\left[\frac{\max Q_{C_{BA}}-Q_{C_{BA}}}{\max Q_{C_{BA}}-\min Q_{C_{BA}}}\right].\left(\frac{LPOC}{LPOC+K_{LPOC}}\right)$ | Equation 22 |

The parameter up_part represents the carbon uptake fraction of bacteria on particulate organic matter.

## Bacterial nitrogen

| $\frac{\partial N_{BA}}{\partial t}=V_{N_{BA}}.NBA-\frac{BP.N_{BA}}{NBA}$ | Equation 23 |
| --- | --- |
| $V_{N_{BA}}=(1-coeff_{BA}).V_{LDON}^{BA}+coeff_{BA}.V_{NH_{4}}^{BA}+V_{DPON}^{BA}$ | Equation 24 |
| $V_{LDON}^{BA}=(1-up\_part).\max V_{LDON}^{BA}.\left[\frac{\max Q_{N_{BA}}-Q_{N_{BA}}}{\max Q_{N_{BA}}-\min Q_{N_{BA}}}\right].\left(\frac{LDON}{LDON+K_{LDON}^{BA}}\right)$ | Equation 25 |
| $V_{NH_{4}}^{BA}=(1-up\_part).\max V_{NH_{4}}^{BA}.\left[\frac{\max Q_{N_{BA}}-Q_{N_{BA}}}{\max Q_{N_{BA}}-\min Q_{N_{BA}}}\right].\left(\frac{NH_{4}}{NH_{4}+K_{NH_{4}}^{BA}}\right)$ | Equation 26 |
| $V_{DPON}^{BA}=up\_part.(coeff_{BA}.V_{LDON}^{BA}+(1-coeff_{BA}).V_{NH_{4}}^{BA})$ | Equation 27 |

## Bacterial phosphorus

| $\frac{\partial P_{BA}}{\partial t}=V_{P_{BA}}.NBA-\frac{BP.P_{BA}}{NBA}$ | Equation 28 |
| --- | --- |
| $V_{P_{BA}}=(1-coeff_{BA}).V_{LDOP}^{BA}+coeff_{BA}.V_{PO_{4}}^{BA}+V_{DPOP}^{BA}$ | Equation 29 |
| $V_{LDOP}^{BA}=(1-up\_part).\max V_{LDOP}^{BA}.\left[\frac{\max Q_{P_{BA}}-Q_{P_{BA}}}{\max Q_{P_{BA}}-\min Q_{P_{BA}}}\right].\left(\frac{LDOP}{LDOP+K_{LDOP}^{BA}}\right)$ | Equation 30 |
| $V_{PO_{4}}^{BA}=(1-up\_part).\max V_{PO_{4}}^{BA}.\left[\frac{\max Q_{P_{BA}}-Q_{P_{BA}}}{\max Q_{P_{BA}}-\min Q_{P_{BA}}}\right].\left(\frac{PO_{4}}{PO_{4}+K_{PO_{4}}^{BA}}\right)$ | Equation 31 |
| $V_{DPOP}^{BA}=up\_part.(coeff_{BA}.V_{LDOP}^{BA}+(1-coeff_{BA}).V_{PO_{4}}^{BA})$ | Equation 32 |

# Detrital particulate organic matter

## Detrital particulate organic carbon (DPOC)

| $\begin{matrix} \frac{\partial DPOC}{\partial t}=-V_{DPOC}^{BA}.NBA+(1-d_{C}).\left(g.f(T).(C_{B}+DPOC)+\frac{BP.C_{BA}}{NBA}\right) \\ .\ldots\ldots\ldots-\left[g.f(T).DPOC\right]+\left[(1-r_{zoo}). d_{C}.k2_{C}.\left(g.f(T).(C_{B}+DPOC)+\frac{BP.C_{BA}}{NBA}\right)\right] \end{matrix}$ | Equation 33 |
| --- | --- |

## Detrital particulate organic nitrogen (DPON)

| $\begin{matrix} \frac{\partial DPON}{\partial t}=-V_{DPON}^{BA}.NBA+(1-d_{N}).\left(g.f(T).(N_{B}+DPON)+\frac{BP.N_{BA}}{NBA}\right) \\ ...........-\left[g.f(T).DPON\right]+\left[d_{N}.k2_{N}.\left(g.f(T).(N_{B}+DPON)+\frac{BP.N_{BA}}{NBA}\right)\right] \end{matrix}$ | Equation 34 |
| --- | --- |

## Detrital particulate organic phosphorus (DPOP)

| $\begin{matrix} \frac{\partial DPOP}{\partial t}=-V_{DPOP}^{BA}.NBA+(1-d_{P}).\left(g.f(T).(P_{B}+DPOP)+\frac{BP.P_{BA}}{NBA}\right) \\ ...........-\left[g.f(T).DPOP\right]+\left[d_{P}.k2_{P}.\left(g.f(T).(P_{B}+DPOP)+\frac{BP.P_{BA}}{NBA}\right)\right] \end{matrix}$ | Equation 35 |
| --- | --- |

# Labile dissolved organic matter

## Labile dissolved organic carbon (LDOC)

| $\begin{matrix} \frac{\partial LDOC}{\partial t}=+(1-r_{Zoo}).d_{C}.(1-k2_{C}).\left(g.f(T).(C_{B}+DPOC)+\frac{BP.C_{BA}}{NBA}\right) \\ \ldots\ldots\ldots\ldots-V_{LDOC}^{BA}.NBA+exu_{LDOC}.P^{C}.C_{B} \end{matrix}$ | Equation 36 |
| --- | --- |

## Labile dissolved organic nitrogen (LDON)

| $\begin{matrix} \frac{\partial LDON}{\partial t}=(1-exu\_nh_{ZOO}).d_{N}.(1-k2_{N}).\left(g.f(T).(N_{B}+DPON)+\frac{BP.N_{BA}}{NBA}\right) \\ ................-(1-coeff_{BA}).V_{LDON}^{BA}.NBA \end{matrix}$ | Equation 37 |
| --- | --- |

## Labile dissolved organic phosphorus (LDOP)

| $\begin{matrix} \frac{\partial LDOP}{\partial t}=(1-exu\_nh_{ZOO}).d_{P}.(1-k2_{P}).\left(g.f(T).(P_{B}+DPOP)+\frac{BP.P_{BA}}{NBA}\right) \\ ................-(1-coeff_{BA}).V_{LDOP}^{BA}.NBA \end{matrix}$ | Equation 38 |
| --- | --- |

# Nutrients, oxygen

## NH_4_

| $\begin{matrix} \frac{\partial NH_{4}}{\partial t}=-V_{NH_{4}}^{C}.C_{B}+exu\_nh_{ZOO}.d_{N}.(1-k2_{N}).\left(g.f(T).(N_{B}+DPON)+\frac{BP.N_{BA}}{NBA}\right) \\ \ldots\ldots...\ldots-coeff_{BA}.V_{NH_{4}}^{BA}.NBA-nit.NH_{4} \end{matrix}$ | Equation 39 |
| --- | --- |

With the nitrification term:

| $nit=nit_{\max}.\exp(k_{Q10}.T).\frac{O_{2}}{O_{2}+K_{O_{2}}}$ | Equation 40 |
| --- | --- |

## NO_3_

| $\frac{\partial NO_{3}}{\partial t}=-V_{NO_{3}}^{C}.C_{B}+nit.NH_{4}$ | Equation 41 |
| --- | --- |

## PO_4_

| $\begin{matrix} \frac{\partial PO_{4}}{\partial t}=-V_{PO_{4}}^{C}.C_{B}+exu\_nh_{ZOO}.d_{P}.(1-k2_{P}).\left(g.(P_{B}+DPOP)+\frac{BP.P_{BA}}{NBA}\right) \\ \ldots\ldots\ldots-coeff_{BA}.V_{PO_{4}}^{BA}.NBA \end{matrix}$ | Equation 42 |
| --- | --- |

## Oxygen

| $\frac{\partial O}{\partial t}=P^{C}.C_{B}-R.C_{B}-2.nit.NH_{4}+2.V_{NO_{3}}^{C}.C_{B}$ | Equation 43 |
| --- | --- |
