# Supplementary material for: Development of a 3D Coupled Physical-Biogeochemical Model for the Marseille Coastal Area (NW Mediterranean Sea): What Complexity Is Required in the Coastal Zone?
Source: PLoS One. 2013 Dec 4;8(12):e80012. doi: 10.1371/journal.pone.0080012 (PMC3851166; doi:10.1371/journal.pone.0080012)
Supplement: Annex S2 — Parameters. (PDF) [file pone.0080012.s002.pdf]

Annex S2. Parameters

Table 1. Parameters, part one

Parameter	Values	Units	Definition	References
m_1	0.43	*d.l.*.	Fraction of the solar energy flux photosynthetically available	(Tett, 1987)
m_2	0.95	*d.l.*	Sea surface reflection	(Tett, 1987)
m_3	0.75	*d.l.*	More rapid attenuation of polychromatic light near the sea surface	(Tett, 1987)
k_0	0.0384	m^{-1}	Extinction factor of sea water	(Lorenzen, 1972)
$A_{Chl.a}$	0.01	m^2 (mg Chl.*a*)$^{-1}$	Chlorophyll absorption factor	(Tett, 1987)
nit_{max}	5.79E-07	s^{-1}	Maximum nitrification rate at 0°C	(Tett, 1990)
K_{O2}	30	mmolO_2 m^{-3}	Half-saturation constant for Oxygen	(Tett, 1990)
k_{Q10}	0.0693	$(°C)^{-1}$	Temperature coefficient for nitrification	(Pinazo et al., 2004; Faure et al., 2010)

Table2. Parameters, part two (Phytoplankton)

Parameter	Values	Units	Definition	References
exu_{LDOC}	*20%*	*d.l*	carbon exudation fraction of primary production	(Faure et al., 2010)
g	9.57E-06	s^{-1}	grazing rate	calibration
r	1.15E-06	s^{-1}	phytoplankton respiration	(Faure et al., 2010)
$alpha\ chl$	8.50E-06	m^2 mmolC (mg Chl.*a* J)$^{-1}$	Chlorophyll-specific light absorption coefficient	(Faure et al., 2010)
a	9.38E-06	s^{-1}	phytoplankton maximum growth rate	(Bissinger et al., 2008)
b	*0.0631*	$(°C)^{-1}$	temperature factor for phytoplankton maximum growth rate	(Bissinger et al., 2008)
$min\ Q^N{}_C$	0.05	mmolN (mmolC)$^{-1}$	Minimum phytoplankton N:C ratio	(Caperon, J., Meyer, 1972)
$max\ Q^N{}_C$	0.25	mmolN (mmolC)$^{-1}$	Maximum phytoplankton N:C ratio	(Caperon, J., Meyer, 1972)
$min\ Q^P{}_C$	0.005	mmolP (mmolC)$^{-1}$	Maximum phytoplankton P:C ratio	(Herrmann, 2007)
$max\ Q^P{}_C$	0.02	mmolP (mmolC)$^{-1}$	Maximum phytoplankton P:C ratio	(Herrmann, 2007)
$max\ V^C{}_{NH4}$	1.10E-05	mmolN (mmolC)$^{-1}$ s^{-1}	Maximum carbon specific uptake rate for NH_4	(Pinazo et al., 1996)
$max\ V^C{}_{NO3}$	4.50E-06	mmolN (mmolC)$^{-1}$ s^{-1}	Maximum carbon specific uptake rate for NO_3	(Pinazo et al., 1996)
$max\ V^C{}_{PO4}$	1.00E-06	mmolP (mmolC)$^{-1}$ s^{-1}	Maximum carbon specific uptake rate for PO4	calibration
K_{NH4}	0.2	mmolN m^{-3}	Half saturation constant for NH_4	(Tett, 1987)
K_{PO4}	0.05	mmolP.m-3	Half saturation constant for po4	(Campbell et al., 2013)
K_{NO3}	0.3	mmolN m^{-3}	Half saturation constant for NO_3	(Tett, 1987)
$max\ Q^{Chl}{}_N$	*2*	mg Chl.*a* (mmolN)$^{-1}$	Maximum Chl.*a*:N ratio	(Smith and Tett, 2000)
$min\ Q^{Chl}{}_N$	1	mg Chl.*a* (mmolN)$^{-1}$	Minimum Chl.*a*:N ratio	(Smith and Tett, 2000)
W_{phyto}	7.50E-06	m.s^{-1}	Sinking rate of phytoplancton	(Pinazo et al., 1996)

Table 3. Parameters, part three (Bacteria)

Parameter	Values	Units	Definition	References
NBA	0.7	10^{12} cell.m^{-3}	Bacterial abundance considered as constant	assumed
μ^{BA}_{max}	$3.6.10^{-5}$	s^{-1}	Maximum growth rate	(Faure et al., 2010)
$minQ^{C}_{BA}$	0.49	mmolC (10^{12} cell)$^{-1}$	Minimum carbon:cell quota	(Fukuda et al., 1998)
max Q^{C}_{BA}	1.95	mmolC (10^{12} cell)$^{-1}$	Maximum carbon:cell quota	(Fukuda et al., 1998)
min Q^{N}_{BA}	0.09	mmolN (10^{12} cell)$^{-1}$	Minimum nitrogen:cell quota	(Fukuda et al., 1998)
max Q^{N}_{BA}	0.23	mmolN (10^{12} cell)$^{-1}$	Maximum nitrogen:cell quota	(Fukuda et al., 1998)
min Q^{P}_{BA}	0.005	mmolP (10^{12} cell)$^{-1}$	Minimum phosphorus:cell quota	(Thingstad, 1987), calibration
max Q^{P}_{BA}	0.02	mmolP (10^{12} cell)$^{-1}$	Minimum phosphorus:cell quota	(Thingstad, 1987)
max V^{BA}_{LDOC}	6.38E-05	mmol C (10^{12} cell)$^{-1}$ h^{-1}	Maximum LDOC specific uptake rate	(Faure et al., 2010)
K_{LDOC}	10	mmol C.m^{-3}	Half-saturation constant carbon	(Faure et al., 2010)
ρ^{g}_{BA}	0.6	*d.l.*	Proportionality factor between carbon incorporated and respired for growth	(Thingstad, 1987)
ρ^{r}_{BA}	0.01	*d.l.*	Fraction of surplus carbon respired	(Thingstad, 1987)
max V^{BA}_{LDON}	3.72E-06	mmol N (10^{12} cell)$^{-1}$ s^{-1}	Maximum LDON specific uptake rate	(Faure et al., 2010)
max V^{BA}_{NH4}	3.72E-06	mmol N (10^{12} cell)$^{-1}$ s^{-1}	Maximum NH_4 specific uptake rate	(Faure et al., 2010)
K^{BA}_{LDON}	1	mmol N.m^{-3}	Half-saturation constant LDON	Data from Torréton 2000-2002
K^{BA}_{NH4}	1	mmol N.m^{-3}	Half-saturation constant NH_4	Data from Torréton 2000-2002
max V^{BA}_{LDOP}	5.55E-06	mmol P (10^{12} cell)$^{-1}$ s^{-1}	Maximum LDOP specific uptake rate	(Thingstad, 1987)
max V^{BA}_{PO4}	5.55E-06	mmol P (10^{12} cell)$^{-1}$ s^{-1}	Maximum PO_4 specific uptake rate	(Thingstad, 1987)
K^{BA}_{PO4}	0.007	mmol P.m^{-3}	Half-saturation constant PO_4	(Campbell et al., 2013)
$coeff_{BA}$	0.2	*d.l.*	DIM/LDOM uptake coefficient	(Harmon, R., Challenor, 1997)
up_part	15%	*d.l.*	Percentage of attached bacteria	(Ghiglione et al., 2007; Mével et al., 2008)

Table 4. Parameters, part four (Zooplancton)

Parameter	Values	Units	Definition	References
d_C	92%	*d.l*	Rendement d'assimilation du carbone	(Gerber, R.P., Gerber, 1979)
				(Le Borgne, R., Blanchot, J., Charpy, 1989)
d_N	95%	*d.l*	Rendement d'assimilation de l'azote	(Faure et al., 2010)
dP	95%	*d.l*	Rendement d'assimilation du phosphore	calibration
r_{ZOO}	31%	*d.l*	Respiration	(Dagg, 1976)
				(Kremer, 1977)
				(Copping, A.E., Lorenzen, 1980)
				(Faure et al., 2010)
				(Small, L.F., Fowler, S.W., Moore, S.A., La Rosa, 1983)
				(Lynch, M., Weider, L.J., Lampert, 1986)
				(Steinberg et al., 2000)
$k2_C$	0.24	*d.l*	Net carbon growth efficiency	(Gerber, R.P., Gerber, 1979)
				(Le Borgne, R., Blanchot, J., Charpy, 1989)
				(Faure et al., 2010)
$k2_N$	0.445	*d.l*	Net nitrogen growth efficiency	(Le Borgne and Rodier, 1997)
$k2p$	0.373	*d.l*	Net phosphorus growth efficiency	(Le Borgne, 1982)
exu_nh_{ZOO}	50%	*d.l*	Fraction excretion term NH4/LDON	(Faure et al., 2010)
				(Le Borgne and Rodier, 1997)
exu_p_{ZOO}	50%	*d.l*	Fraction excretion term PO4/LDOP	calibration

References:

Bissinger, J.E., Montagnes, D.J.S., Sharples, J., 2008. Predicting marine phytoplankton maximum growth rates from temperature: Improving on the Eppley curve using quantile regression 53, 487–493.

Campbell, R., Diaz, F., Hu, Z., Doglioli, A., Petrenko, A., Dekeyser, I., 2013. Nutrients and plankton spatial distributions induced by a coastal eddy in the Gulf of Lion. Insights from a numerical model. Progress in Oceanography 109, 47–69.

Caperon, J., Meyer, J., 1972. Nitrogen-limited growth of marine phytoplankton. II. Uptake kinetic and their role in nutrient growth of phytoplankton. Deep-Sea Research Part I – Oceanographic Research Papers 19, 619–632.

Copping, A.E., Lorenzen, C.J., 1980. Carbon budget of a marine phytoplankton- herbivore system with carbon-14 as a tracer. Limnology and Oceanography 25, 873–882.

Dagg, M.J., 1976. Complete carbon and nitrogen budgets for the carnivorous amphipod, Calliopius laeviusculus (Kroyer). Internationale Revue der Gesamten Hydrobiologie 61, 297–357.

Faure, V., Pinazo, C., Torreton, J.-P., Jacquet, S., 2010. Modelling the spatial and temporal variability of the SW lagoon of New Caledonia I: a new biogeochemical model based on microbial loop recycling. Marine pollution bulletin 61, 465–79.

Fukuda, R., Ogawa, H., Nagata, T., Koike, I., 1998. Direct determination of carbon and nitrogen contents of natural bacterial assemblages in marine environments. Applied and environmental microbiology 64, 3352–8.

Gerber, R.P., Gerber, M.B., 1979. Ingestion of natural particulate organic matter and subsequent assimilation, respiration and growth by tropical lagoon zooplankton. Marine Biology 52, 33–43.

Ghiglione, J.F., Mevel, G., Pujo-Pay, M., Mousseau, L., Lebaron, P., Goutx, M., 2007. Diel and seasonal variations in abundance, activity, and community structure of particle-attached and free-living bacteria in NW Mediterranean Sea. Microbial ecology 54, 217–31.

Harmon, R., Challenor, P., 1997. A Markov chain Monte Carlo method for estimation and assimilation into models. Ecological Modelling 101, 41–59.

Herrmann, M., 2007. Formation et devenir des masses d'eau en Méditerranée nord-occidentale Influence sur l'écosystème planctonique pélagique Variabilité interannuelle et changement climatique. 2007.

Kremer, P., 1977. Respiration and excretion by the ctenophore Mnepiopsis leidyi. Marine Biology 44, 43–50.

Le Borgne, R., Rodier, M., 1997. Net zooplankton and the biological pump: a comparison between the oligotrophic and mesotrophic equatorial Pacific. Deep Sea Research Part II: Topical Studies in Oceanography 44, 2003–2023.

Le Borgne, R., Blanchot, J., Charpy, L., 1989. Zooplankton of the atoll of Tikehau (Tuamotu Archipelago) and its relations with particulate matter. Marine Biology 102, 341–353.

Lorenzen, C.J., 1972. Extinction of light in the ocean by phytoplankton. Journal du Conseil Permanent International pour l'Exploration de la Mer 34, 262–267.

Lynch, M., Weider, L.J., Lampert, W., 1986. Measurement of the carbon balance in Daphnia. Limnology and Oceanography 31, 17–33.

Mével, G., Vernet, M., Goutx, M., Ghiglione, J.F., 2008. Seasonal to hour variation scales in abundance and production of total and particle-attached bacteria in the open NW Mediterranean Sea (0–1000 m). Biogeosciences 5, 1573–1586.

Pinazo, C., Bujan, S., Douillet, P., Fichez, R., Grenz, C., Maurin, a., 2004. Impact of wind and freshwater inputs on phytoplankton biomass in the coral reef lagoon of New Caledonia during the summer cyclonic period: a coupled three-dimensional biogeochemical modeling approach. Coral Reefs 23, 281–296.

Pinazo, C., Marsaleix, P., Millet, B., Estournel, C., Véhil, R., 1996. Spatial and temporal variability of phytoplankton biomass in upwelling areas of the northwestern mediterranean: a coupled physical and biogeochemical modelling approach. Journal of Marine Systems 7, 161–191.

Small, L.F., Fowler, S.W., Moore, S.A., La Rosa, J., 1983. Dissolved and fecal pellet carbon and nitrogen release by zooplankton in tropical waters. Deep-Sea Research Part I – Oceanographic Research Papers 30, 1199–1220.

Smith, C.L., Tett, P., 2000. A depth-resolving numerical model of physically forced microbiology at the European shelf edge. Journal of Marine Systems 26, 1–36.

Steinberg, D.K., Carlson, C. a., Bates, N.R., Goldthwait, S. a., Madin, L.P., Michaels, A.F., 2000. Zooplankton vertical migration and the active transport of dissolved organic and inorganic carbon in the Sargasso Sea. Deep Sea Research Part I: Oceanographic Research Papers 47, 137–158.

Tett, P., 1987. Modelling the growth and distribution of marine microplankton. Proceedings of the Society for General Microbiology Symposium 41 ,“Ecology of Microbial Communities” 41, 387–425.

Tett, P., 1990. A Three Layer Vertical and Microbiological Processes Model for Shelf Seas. Proudman Oceanographic Laboratory, Birkenhead Report No., 85.

Thingstad, T.F., 1987. Utilization of N, P, and organic C by heterotrophic bacteria. I Outline of a chemostat theory with a consistent concept of “maintenance” metabolism. Marine Ecology Progress Series 35, 99– 109.
