# Supplementary material for: Development of a 3D Coupled Physical-Biogeochemical Model for the Marseille Coastal Area (NW Mediterranean Sea): What Complexity Is Required in the Coastal Zone?
Source: PLoS One. 2013 Dec 4;8(12):e80012. doi: 10.1371/journal.pone.0080012 (PMC3851166; doi:10.1371/journal.pone.0080012)

Annex S3 . Spin up of the MARS3D-RHOMA /ECO3M-MASSILIA coupled model for the year 2008 at the Somlit station at the surface.
“Model 1” corresponding to the summer initial conditions and “Model 2” corresponding to the winter initial conditions.


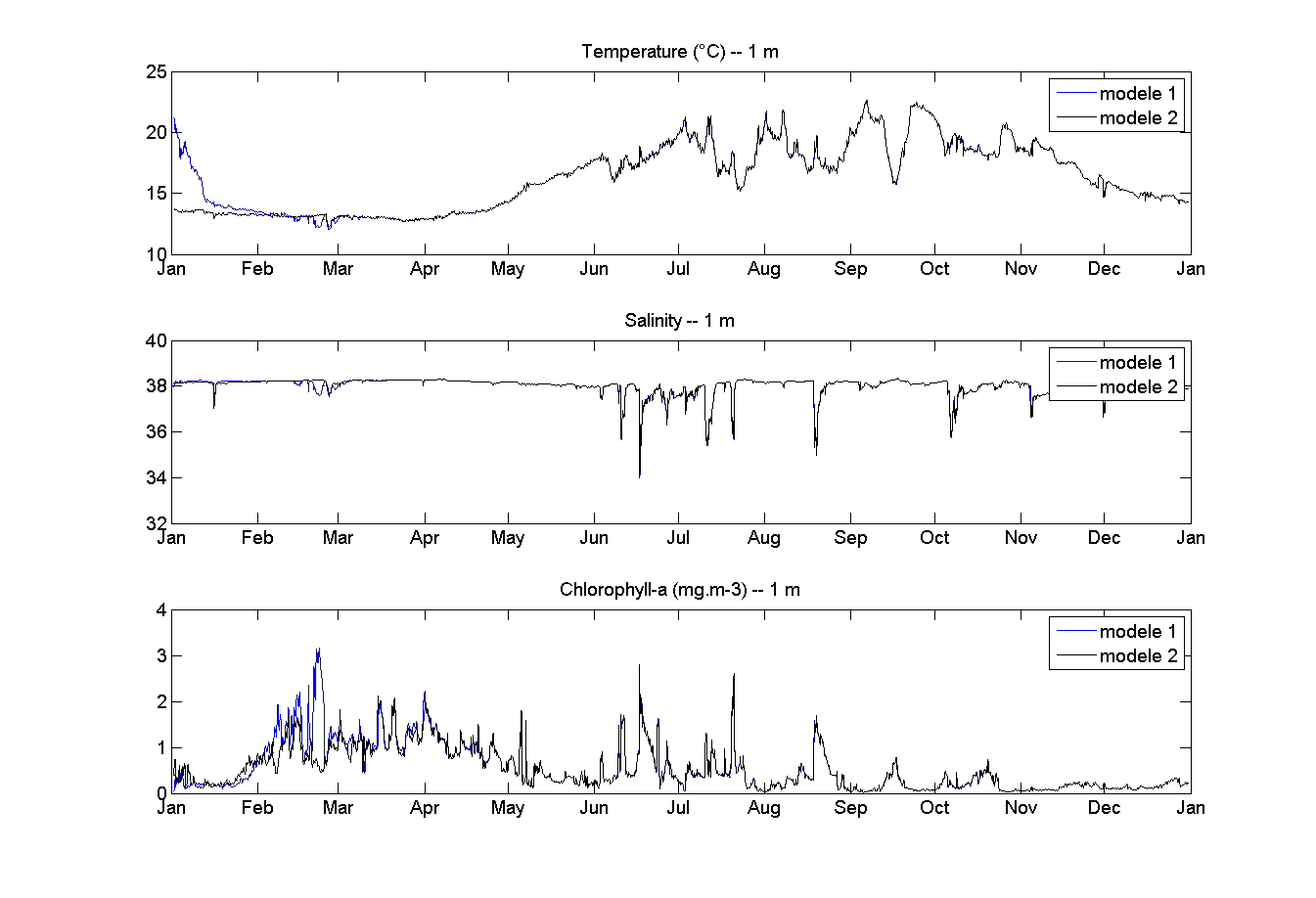


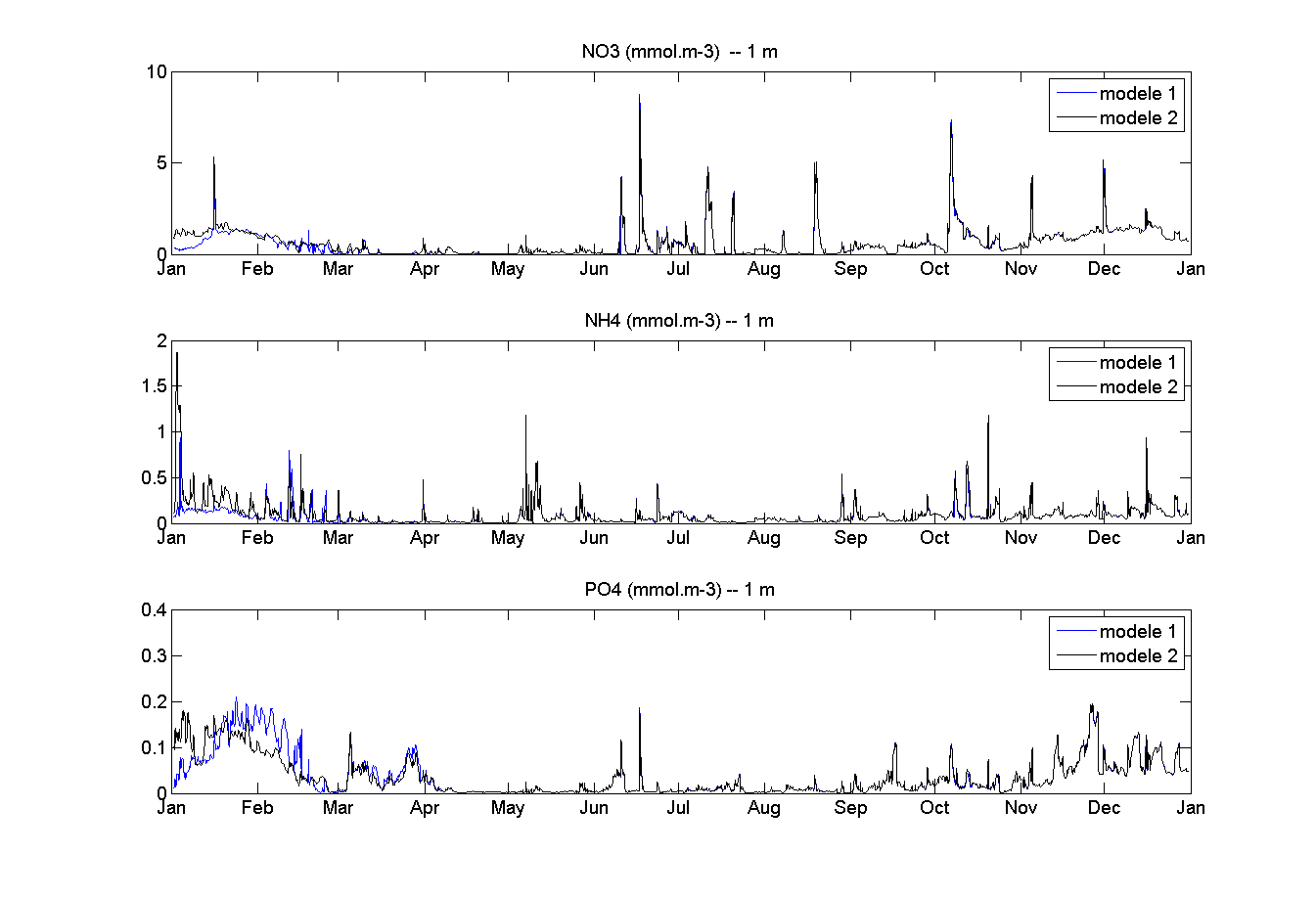

Supplement: Annex S3 — Spin up of the MARS3D-RHOMA/ECO3M-MASSILIA coupled model for the year 2008 at the Somlit station at the surface. “Model 1” corresponding to the summer initial conditions and “Model 2” corresponding to the winter initial conditions. (DOCX) [file pone.0080012.s003.docx]
