# Supplementary figures and images for: Development of a 3D Coupled Physical-Biogeochemical Model for the Marseille Coastal Area (NW Mediterranean Sea): What Complexity Is Required in the Coastal Zone?
Source: PLoS One. 2013 Dec 4;8(12):e80012. doi: 10.1371/journal.pone.0080012 (PMC3851166; doi:10.1371/journal.pone.0080012)

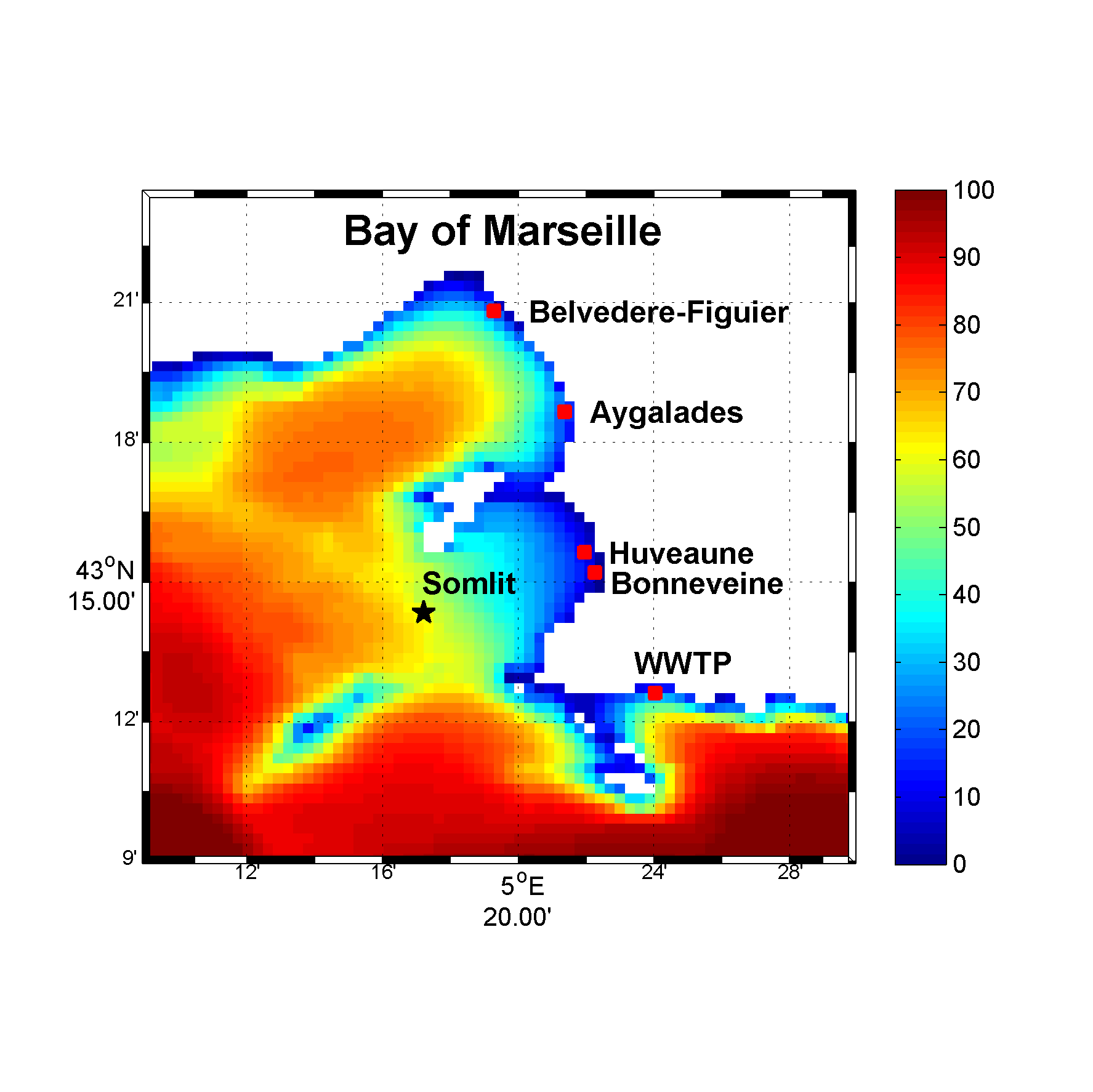

Supplement: Annex S4 — Maps of the Marseille city inputs. (TIF) [file pone.0080012.s004.tif]
