# Supplementary material for: Development of a 3D Coupled Physical-Biogeochemical Model for the Marseille Coastal Area (NW Mediterranean Sea): What Complexity Is Required in the Coastal Zone?
Source: PLoS One. 2013 Dec 4;8(12):e80012. doi: 10.1371/journal.pone.0080012 (PMC3851166; doi:10.1371/journal.pone.0080012)
Supplement: Annex S5 — Quality of the remote sensing data. (DOCX) [file pone.0080012.s005.docx]

**Annex S5: Quality of the remote sensing data**

The temporal resolution is on the order of days, but cloud cover usually obstructs at least part of the study region. However, the Marseille coastal area is generally sunny; therefore, there is good coverage by the optical ocean color sensors. From 2007 to 2011, 754 and 509 daily chlorophyll-a concentrations were available at the Somlit station for the MODIS and MERIS sensors, respectively; thus, 40 % of the time, chlorophyll-a data were available from MODIS, and 27 % of the time from MERIS. During these 5 years, the MODIS and MERIS data were, respectively, 21.1 % and 23.8 % available in winter (December-January-February), 23.6 % and 23.4 % in spring (March-April-May), 29.7 % and 24.4 % in summer (June-July-August) and 25.6 % and 28.5 % in fall (September-October-November). Therefore, there was good repetition of the remote sensing data over the seasons.

Table: Statistical indicators of the remote sensing data versus the in-situ data of chlorophyll-a at the Somlit station (2007-2011)

|  | MODIS | MERIS |
| --- | --- | --- |
| n | 52 | 33 |
| Mean (in-situ) | 0.35 | 0.4 |
| Mean (remote sensing) | 0.36 | 0.36 |
| Std (in-situ) | 0.18 | 0.4 |
| Std (remote sensing) | 0.3 | 0.19 |
| CF | 0.87 | 0.45 |
| Bias (%) | -2.58 | 10.73 |
| AAE | 0.15 | 0.17 |
| RMSD | 0.27 | 0.27 |
| R | 0.45 | 0.81 |

The quality of the remote sensing data was assessed by comparing them with the Somlit station in-situ surface chlorophyll-a concentrations (Table). The average absolute error for the chlorophyll-a concentration between the in-situ and remote sensing data was 0.15 and 0.17 µg.L^-1^ for MODIS and MERIS, respectively. We observed that the accuracies of the two sensors in representing the in-situ data differed. Data from MERIS (R=0.85) were better correlated with the in-situ data than those from MODIS (R=0.45), whereas the MODIS bias (<3 %) was very small in contrast with the underestimation by MERIS of the chlorophyll-a concentration by approximately 10 %.
